# Correction: It’s not all abundance: Detectability and accessibility of food also explain breeding investment in long-lived marine animals

**DOI:** 10.1371/journal.pone.0329667

**Published:** 2025-08-04

**Authors:** The PLOS One Staff

## Notice of Republication

This article was republished on July 10, 2025, to correct an error in the author list: Daniel Oro was erroneously displayed as Daniel Orol. Additionally, there was an error in affiliation 1 for authors Enric Real, Daniel Oro, José Manuel Igual, Ana Sanz-Aguilar, Meritxell Genovart, and Giacomo Tavecchia. The correct affiliation 1 is: Animal Demography and Ecology Unit, Instituto Mediterráneo de Estudios Avanzados, Esporles, Spain. The publisher apologizes for the errors. Please download this article again to view the correct version. The originally published, uncorrected article and the republished, corrected articles are provided here for reference.

## Supporting information

S1 File Originally published, uncorrected article. (PDF)

S2 File Republished, corrected article. (PDF)
