# Supplementary material for: It’s not all abundance: Detectability and accessibility of food also explain breeding investment in long-lived marine animals
Source: PLoS One. 2022 Sep 21;17(9):e0273615. doi: 10.1371/journal.pone.0273615 (PMC9491606; doi:10.1371/journal.pone.0273615)
Supplement: S1 Table — (DOCX) [file pone.0273615.s001.docx]

S1 Table. Generalized linear models explaining egg volume variability (mean egg volume in a clutch) of the Scopoli’s shearwater based on Akaike information criterion values (AIC) and Akaike weights (Wi). The best explanatory model (Model 1) is the one with the lowest AIC. In the notation: Winter NAO = winter North Atlantic Oscillation, Spring NAO = Spring North Atlantic Oscillation considering the species-specific pre-laying period, SS = Scopoli’s shearwater, YLG = Yellow-legged Gull, AG = Audouin’s Gull, PC= per capita, Wind1Q, 2Q, 3Q and 4Q = 1^st^ ,2^nd^ ,3^rd^ and 4^th^ quartile winds respectively (see methods section), Discards = fishery discards, Null model is an only-intercept model. Discards PC and Sardine PC consider the number of individuals of YLG+AG+SS.

| Model | Notation | Deviance | df | AIC | ∆AIC | Wi |
| --- | --- | --- | --- | --- | --- | --- |
| 1 | Winter NAO + WaveHeight * DiscardsPC | 40733.54 | 6 | 8911.61 | 0.00 | 0.85 |
| 2 | Winter NAO + WaveHeight + DiscardsPC | 40899.95 | 5 | 8915.48 | 3.87 | 0.12 |
| 3 | WaveHeight * DiscardsPC | 40966.80 | 5 | 8917.83 | 6.22 | 0.04 |
| 4 | Winter NAO + WaveHeight | 41035.65 | 4 | 8918.25 | 6.88 | 0.03 |
| 5 | Winter NAO | 41331.38 | 3 | 8926.59 | 14.98 | 0.00 |
| 6 | WaveHeight | 41478.83 | 3 | 8931.72 | 20.11 | 0.00 |
| 7 | Competition by AG | 41558.13 | 3 | 8934.47 | 22.86 | 0.00 |
| 8 | Wind4Q | 41602.25 | 3 | 8936.00 | 24.39 | 0.00 |
| 9 | Discards PC | 41635.50 | 3 | 8937.15 | 25.54 | 0.00 |
| 10 | Wind3Q | 41693.72 | 3 | 8939.16 | 27.55 | 0.00 |
| 11 | Wind1Q | 41734.33 | 3 | 8940.56 | 28.95 | 0.00 |
| 12 | Sardine PC | 41754.58 | 3 | 8941.26 | 29.65 | 0.00 |
| 13 | Wind2Q | 41979.94 | 3 | 8949.01 | 37.40 | 0.00 |
| 14 | Turbidity | 42004.55 | 3 | 8949.86 | 38.25 | 0.00 |
| 15 | NullModel | 42112.58 | 2 | 8951.56 | 39.95 | 0.00 |
| 16 | Spring NAO | 42060.46 | 3 | 8951.77 | 40.16 | 0.00 |
| 17 | Intraspecific competition | 42107.99 | 3 | 8953.40 | 41.79 | 0.00 |
| 18 | Competition by YLG | 42112.14 | 3 | 8953.54 | 41.93 | 0.00 |
