# Supplementary material for: It’s not all abundance: Detectability and accessibility of food also explain breeding investment in long-lived marine animals
Source: PLoS One. 2022 Sep 21;17(9):e0273615. doi: 10.1371/journal.pone.0273615 (PMC9491606; doi:10.1371/journal.pone.0273615)
Supplement: S2 Table — (DOCX) [file pone.0273615.s002.docx]

S2 Table. Generalized linear models explaining egg volume variability (mean egg volume in a clutch) of the Sandwich tern based on Akaike information criterion values (AIC) and Akaike weights (Wi). The best explanatory model (Model 1) is the one with the lowest AIC. In the notation: Winter NAO = winter North Atlantic Oscillation, Spring NAO = Spring North Atlantic Oscillation considering the species-specific pre-laying period, YLG = Yellow-legged Gull , PC= Per capita, Wind1Q, 2Q, 3Q and 4Q = 1^st^ ,2^nd^ ,3^rd^ and 4^th^ quartile winds respectively (see methods section), Discards = fishery discards, Null model is an only-intercept model. Both, discards PC and anchovy PC consider the number of individuals of ST, YLG, AG and SS.

| Model | Notation | Deviance | df | AIC | ∆AIC | Wi |
| --- | --- | --- | --- | --- | --- | --- |
| 1 | Winter NAO + Wind3Q + Turbidity | 1655.94 | 5 | 1787.71 | 0.00 | 0.23 |
| 2 | Winter NAO + Wind3Q | 1665.30 | 4 | 1788.10 | 0.39 | 0.19 |
| 3 | Winter NAO + Wind3Q + Turbidity + Competition by YLG | 1650.57 | 6 | 1788.34 | 0.63 | 0.17 |
| 4 | Winter NAO + Wind3Q + Competition by YLG | 1662.09 | 5 | 1789.28 | 1.57 | 0.10 |
| 5 | Winter NAO + Wind3Q + AnchovyPC | 1659.32 | 5 | 1789.58 | 1.87 | 0.08 |
| 6 | Winter NAO + Wind3Q * Turbidity | 1655.69 | 6 | 1789.65 | 1.94 | 0.09 |
| 7 | Winter NAO + Wind3Q + Wind1Q | 1664.41 | 5 | 1789.87 | 2.16 | 0.08 |
| 8 | Winter NAO | 1689.33 | 3 | 1792.16 | 4.45 | 0.02 |
| 9 | Wind3Q | 1690.22 | 3 | 1792.38 | 4.67 | 0.02 |
| 10 | Wind3Q + Turbidity | 1689.84 | 4 | 1794.30 | 6.59 | 0.01 |
| 11 | Spring NAO | 1704.32 | 3 | 1795.89 | 8.18 | 0.00 |
| 12 | Wave height | 1708.91 | 3 | 1797.03 | 9.32 | 0.00 |
| 13 | Wind1Q | 1709.45 | 3 | 1797.16 | 9.45 | 0.00 |
| 14 | Null model | 1718.36 | 2 | 1797.36 | 9.65 | 0.00 |
| 15 | Competition by YLG | 1711.99 | 3 | 1797.79 | 9.65 | 0.00 |
| 16 | Anchovy PC | 1712.60 | 3 | 1797.94 | 10.23 | 0.00 |
| 17 | Wind4Q | 1713.32 | 3 | 1798.12 | 10.41 | 0.00 |
| 18 | Discards PC | 1713.65 | 3 | 1798.20 | 10.49 | 0.00 |
| 19 | Turbidity | 1715.30 | 3 | 1798.61 | 10.90 | 0.00 |
| 20 | Intraspecific competition | 1715.46 | 3 | 1798.65 | 10.94 | 0.00 |
| 21 | Wind2Q | 1718.35 | 3 | 1799.36 | 11.65 | 0.00 |
|  |  |  |  |  |  |  |
