# Supplementary material for: It’s not all abundance: Detectability and accessibility of food also explain breeding investment in long-lived marine animals
Source: PLoS One. 2022 Sep 21;17(9):e0273615. doi: 10.1371/journal.pone.0273615 (PMC9491606; doi:10.1371/journal.pone.0273615)
Supplement: S3 Table — (DOCX) [file pone.0273615.s003.docx]

S3 Table. Generalized linear models explaining egg volume variability (mean egg volume in a clutch) of the Audouin’s gull based on Akaike information criterion values (AIC) and Akaike weights (Wi). The best explanatory model (Model 1) is the one with the lowest AIC. In the notation: Winter NAO = winter North Atlantic Oscillation, Spring NAO = Spring North Atlantic Oscillation considering the species-specific pre-laying period, YLG = Yellow-legged Gull, AG= Audouin’s Gull, SS= Scopoli’s shearwater, PC= Per capita, Wind1Q, 2Q, 3Q and 4Q = 1^st^ ,2^nd^ ,3^rd^ and 4^th^ quartile winds respectively (see methods section), Discards = fishery discards, Null model is an only-intercept model. Discards PC, Sardine PC and Anchovy PC consider the number of individuals of YLG+AG+SS.

| Model | Notation | Deviance | df | AIC | ∆AIC | W |
| --- | --- | --- | --- | --- | --- | --- |
| 1 | Winter NAO + Intraspecific competition * Competition by YLG | 94498.10 | 6 | 45482.82 | 0.00 | 1.00 |
| 2 | Winter NAO + Intraspecific competition + Competition by YLG | 94640.28 | 5 | 45493.91 | 11.09 | 0.00 |
| 3 | Winter NAO + Intraspecific competition | 94695.19 | 4 | 45496.96 | 14.14 | 0.00 |
| 4 | Intraspecific competition * Competition by YLG | 95377.26 | 5 | 45561.45 | 78.63 | 0.00 |
| 5 | Intraspecific competition | 95560.62 | 3 | 45574.18 | 91.36 | 0.00 |
| 6 | Sardine PC | 95927.54 | 3 | 45607.54 | 124.72 | 0.00 |
| 7 | Wave height | 97782.89 | 3 | 45774.34 | 291.52 | 0.00 |
| 8 | Anchovy PC | 97811.19 | 3 | 45776.86 | 294.04 | 0.00 |
| 9 | Winter NAO | 98076.86 | 3 | 45800.48 | 317.66 | 0.00 |
| 10 | 4^th^ q. winds | 98114.50 | 3 | 45803.82 | 321.00 | 0.00 |
| 11 | Discard PC | 98233.25 | 3 | 45814.35 | 331.53 | 0.00 |
| 12 | 2^nd^ q. winds | 99288.54 | 3 | 45819.25 | 336.43 | 0.00 |
| 13 | Competition by YLG | 99061.17 | 3 | 45887.43 | 404.61 | 0.00 |
| 14 | 1^st^ q. winds | 99109.83 | 3 | 45891.70 | 408.88 | 0.00 |
| 15 | 3^rd^ q. winds | 99719.21 | 3 | 45945.07 | 462.25 | 0.00 |
| 16 | Turbidity | 99871.94 | 3 | 45958.40 | 475.58 | 0.00 |
| 17 | Null | 99945.84 | 2 | 45962.84 | 480.02 | 0.00 |
| 18 | Competition by SS | 99936.80 | 3 | 45964.05 | 481.23 | 0.00 |
| 19 | Spring NAO | 99943.13 | 3 | 45964.60 | 481.78 | 0.00 |
