# Supplementary material for: It’s not all abundance: Detectability and accessibility of food also explain breeding investment in long-lived marine animals
Source: PLoS One. 2022 Sep 21;17(9):e0273615. doi: 10.1371/journal.pone.0273615 (PMC9491606; doi:10.1371/journal.pone.0273615)
Supplement: S4 Table — (DOCX) [file pone.0273615.s004.docx]

S4 Table. Species-specific early breeding periods of studied populations considering from the mean date of arrival to the breeding colony to the mean laying dates. Different fishing fleets considered to estimate abundances of fishery discards and natural prey (see methods in the main manuscript) varies according to the species-specific home range during the breeding season. Sca: Sant Carles, Tar: Tarragona, Ame: Ametlla de Mar, Amp: Amposta, Cal: Cases d’Alcanar, Del: Deltebre.

| Species | Arrival to the breeding colony | Mean laying date | Home range during the breeding season | Fishing ports considered | Home range reference |
| --- | --- | --- | --- | --- | --- |
| Scopoli's shearwater | early March | end of May | 150-200 Km | Ame, Amp, Cal, Del, Sca, Tar | Reyes-González et al 2017 |
| Sandwich tern | early April | end of May | 15 Km | Sca, Tar | Fassola 1990 |
| Audouin's gull | early March | end of April | 70 Km | Ame, Amp, Cal, Del, Sca, Tar | Bécares et al 2015 |
