# Supplementary material for: It’s not all abundance: Detectability and accessibility of food also explain breeding investment in long-lived marine animals
Source: PLoS One. 2022 Sep 21;17(9):e0273615. doi: 10.1371/journal.pone.0273615 (PMC9491606; doi:10.1371/journal.pone.0273615)
Supplement: S5 Table — (DOCX) [file pone.0273615.s005.docx]

S5 Table. In the left side, species considered to compete for the same food resource types in the study area used to assess per capita food abundance (in this case, foraging strategies may be very different and individuals of different species don’t necessarily match in space and time), and in the right side, species considered when considering interspecific interactions during the foraging process (due to species-specific foraging strategies, individuals of different species can match in space and time and interspecific interactions as kleptoparasitism can occur).

| Species | Food resource type | | | Interspecific interactions during the foraging process | | |
| --- | --- | --- | --- | --- | --- | --- |
|  | Sardines | Anchovies | Fishery discards | Scopoli's shearwater | Sandwich tern | Audouin's gull |
| Scopoli's shearwater | x |  | x | x |  | x |
| Sandwich tern |  | x |  |  | x |  |
| Audouin's gull | x | x | x | x |  | x |
| Yellow-legged gull | x | x | x | x | x | x |
