# Supplementary material for: It’s not all abundance: Detectability and accessibility of food also explain breeding investment in long-lived marine animals
Source: PLoS One. 2022 Sep 21;17(9):e0273615. doi: 10.1371/journal.pone.0273615 (PMC9491606; doi:10.1371/journal.pone.0273615)
Supplement: S7 Table — (DOCX) [file pone.0273615.s007.docx]

S7 Table. Correlation matrix based on Pearson’s correlations among all covariates considered for modelling the Sandwich terns breeding investment. Used values corresponds to April (when birds arrive to the breeding colony) to May (mean laying date) means except for the winter NAO (December to March means) for the period 2000-2016 (n=15). Correlations where p-value < 0.05 are shown in bold.

|  | TS  Pairs | Winter  NAO | Spring NAO | PC  anchovy | PC  discards | Wave  Height | 1stQ.  wind | 2ndQ.  wind | 3rdQ.  wind | 4thQ.  wind |
| --- | --- | --- | --- | --- | --- | --- | --- | --- | --- | --- |
| Winter NAO | -0.25 |  |  |  |  |  |  |  |  |  |
| Spring NAO | -0.27 | -0.05 |  |  |  |  |  |  |  |  |
| PCanchovy | 0.05 | 0.09 | 0.24 |  |  |  |  |  |  |  |
| PCdiscards | -0.05 | -0.17 | 0.09 | **-0.64** |  |  |  |  |  |  |
| WaveHeight | 0.24 | 0.39 | -0.28 | 0.04 | -0.3 |  |  |  |  |  |
| 1stQ.wind | -0.39 | **0.53** | 0.07 | -0.03 | -0.24 | 0.4 |  |  |  |  |
| 2ndQ.wind | 0.14 | 0.47 | **-0.59** | 0.11 | -0.18 | **0.67** | 0.36 |  |  |  |
| 3rdQ.wind | 0.06 | 0.17 | -0.35 | 0.14 | **-0.53** | **0.52** | -0.1 | 0.32 |  |  |
| 4thQ.wind | 0.4 | 0.18 | 0.02 | 0.25 | -0.17 | **0.8** | 0.08 | 0.42 | 0.45 |  |
| Turbidity | 0.21 | -0.31 | 0.22 | -0.2 | 0.3 | 0.3 | 0.13 | 0.16 | -0.29 | 0.34 |
