# Supplementary material for: It’s not all abundance: Detectability and accessibility of food also explain breeding investment in long-lived marine animals
Source: PLoS One. 2022 Sep 21;17(9):e0273615. doi: 10.1371/journal.pone.0273615 (PMC9491606; doi:10.1371/journal.pone.0273615)
Supplement: S8 Table — (DOCX) [file pone.0273615.s008.docx]

S8 Table. Correlation matrix based on Pearson’s correlations among all covariates considered for modeling the Audouin’s gull’s breeding investment. Used values corresponds to March (when birds arrive to the breeding colony) to April (mean laying date) means except for the winter NAO (December to March means) for the period 2001-2017 (n=17). Correlations where p-value < 0.05 are shown in bold.

|  | IA  pairs | LM pairs | CD pairs | Winter  NAO | Spring  NAO | PC Sardine | PC Anchovy | PC discards | Wave  Height | 1stQ.  wind | 2ndQ.  wind | 3rdQ.  wind | 4thQ.  wind |
| --- | --- | --- | --- | --- | --- | --- | --- | --- | --- | --- | --- | --- | --- |
| LM pairs | 0.27 |  |  |  |  |  |  |  |  |  |  |  |  |
| CD pairs | -0.01 | -0.42 |  |  |  |  |  |  |  |  |  |  |  |
| wNAO | -0.29 | -0.01 | 0.03 |  |  |  |  |  |  |  |  |  |  |
| Spring NAO | -0.17 | -0.12 | 0.07 | **0.51** |  |  |  |  |  |  |  |  |  |
| PC Sardine | **0.76** | -0.13 | 0.17 | -0.33 | -0.01 |  |  |  |  |  |  |  |  |
| PC Anchovy | **-0.69** | -0.05 | 0.32 | 0.18 | -0.01 | **-0.75** |  |  |  |  |  |  |  |
| PC discards | **0.72** | -0.24 | -0.12 | -0.29 | 0.05 | **0.83** | **-0.85** |  |  |  |  |  |  |
| WaveHeight | **-0.5** | 0.17 | -0.38 | 0.35 | -0.04 | **-0.53** | 0.12 | **-0.49** |  |  |  |  |  |
| 1stQ.wind | -0.4 | 0.24 | -0.36 | **0.49** | 0.26 | **-0.51** | 0.26 | **-0.48** | **0.71** |  |  |  |  |
| 2ndQ.wind | -0.36 | -0.05 | -0.22 | 0.22 | -0.11 | **-0.56** | 0.3 | -0.39 | **0.5** | 0.35 |  |  |  |
| 3rdQ.wind | -0.11 | 0.15 | 0.1 | -0.1 | -0.11 | -0.13 | 0.16 | -0.2 | 0.02 | -0.47 | 0.26 |  |  |
| 4thQ.wind | -0.31 | 0.13 | 0.16 | 0.41 | -0.01 | -0.21 | 0.08 | -0.34 | **0.51** | 0.01 | 0.14 | **0.59** |  |
| Turbidity | -0.22 | -0.31 | -0.13 | -0.22 | -0.35 | -0.02 | -0.04 | 0.04 | 0.33 | 0.11 | -0.2 | -0.33 | -0.05 |
