# Supplementary material for: It’s not all abundance: Detectability and accessibility of food also explain breeding investment in long-lived marine animals
Source: PLoS One. 2022 Sep 21;17(9):e0273615. doi: 10.1371/journal.pone.0273615 (PMC9491606; doi:10.1371/journal.pone.0273615)
Supplement: S9 Table — (DOCX) [file pone.0273615.s009.docx]

S9 Table. Annual values of the observed egg volume for the Scopoli’s shearwater and covariates retained by the best explanatory model (see Model 1 in Tables 1 and S1).

| Year | EggVolumeObserved | wNAO | DiscardsPC | WaveHeight |
| --- | --- | --- | --- | --- |
| 2001 | 74.30 | -1.90 | 0.00075 | 0.65 |
| 2002 | 73.69 | 0.76 | 0.00072 | 0.75 |
| 2003 | 73.99 | 0.20 | 0.00046 | 0.73 |
| 2004 | 73.42 | -0.07 | 0.00047 | 0.71 |
| 2005 | 75.68 | 0.12 | 0.00025 | 0.61 |
| 2006 | 73.81 | 0.12 | 0.00027 | 0.65 |
| 2007 | 72.96 | 2.79 | 0.00026 | 0.77 |
| 2008 | 72.14 | 2.10 | 0.00022 | 0.77 |
| 2009 | 73.85 | -0.41 | 0.00001 | 0.67 |
| 2010 | 74.78 | -4.64 | -0.00010 | 0.65 |
| 2011 | 73.51 | -1.57 | -0.00017 | 0.69 |
| 2012 | 71.24 | 3.17 | -0.00022 | 0.83 |
| 2013 | 72.67 | -1.97 | -0.00035 | 1.02 |
| 2014 | 71.89 | 3.10 | -0.00049 | 0.81 |
| 2015 | 72.38 | 3.56 | -0.00055 | 0.86 |
| 2016 | 72.69 | 0.98 | -0.00065 | 0.88 |
| 2017 | 72.15 | 1.47 | -0.00078 | 0.64 |
