# Supplementary material for: It’s not all abundance: Detectability and accessibility of food also explain breeding investment in long-lived marine animals
Source: PLoS One. 2022 Sep 21;17(9):e0273615. doi: 10.1371/journal.pone.0273615 (PMC9491606; doi:10.1371/journal.pone.0273615)
Supplement: S10 Table — (DOCX) [file pone.0273615.s010.docx]

S10 Table. Annual values of the egg volume observed for the Sadwich tern and covariates retained by the best explanatory model (see Model 1 in Tables 2 and S2). n.a.= data not available.

| Year | EggVolumeObserved | W_NAO | Vient3Q | Kd490 |
| --- | --- | --- | --- | --- |
| 2000 | 34.3 | 2.8 | 180.73 | 0.07 |
| 2001 | n.a. | -1.9 | 179.19 | 0.09 |
| 2002 | 34.0 | 0.76 | 166.61 | 0.07 |
| 2003 | 34.4 | 0.2 | 153.11 | 0.10 |
| 2004 | n.a. | -0.07 | 156.56 | 0.10 |
| 2005 | 34.91 | 0.12 | 127.80 | 0.09 |
| 2006 | n.a. | -1.09 | 165.47 | 0.08 |
| 2007 | 34.3 | 2.79 | 162.72 | 0.07 |
| 2008 | 33.8 | 2.1 | 220.23 | 0.07 |
| 2009 | 34.45 | -0.41 | 203.28 | 0.07 |
| 2010 | n.a. | -4.64 | 200.32 | 0.07 |
| 2011 | 34.7 | -1.57 | 156.45 | 0.06 |
| 2012 | 33.1 | 3.17 | 237.63 | 0.07 |
| 2013 | 34.4 | -1.97 | 212.64 | 0.10 |
| 2014 | n.a. | 3.1 | 210.40 | 0.08 |
| 2015 | 33.9 | 3.56 | 176.65 | 0.07 |
| 2016 | 34.9 | 0.98 | 216.44 | 0.07 |
