# Supplementary material for: It’s not all abundance: Detectability and accessibility of food also explain breeding investment in long-lived marine animals
Source: PLoS One. 2022 Sep 21;17(9):e0273615. doi: 10.1371/journal.pone.0273615 (PMC9491606; doi:10.1371/journal.pone.0273615)
Supplement: S11 Table — (DOCX) [file pone.0273615.s011.docx]

S11 Table. Annual values of the egg volume observed for the Audouin’s gull and covariates retained by the best explanatory model (see Model 1 in Tables 3 and S3).

| Year | Egg Volume Observed | W_NAO | Michahellis Pairs | Audouin Pairs |
| --- | --- | --- | --- | --- |
| 2001 | 57.21 | -1.90 | 3954 | 11666 |
| 2002 | 57.43 | 0.76 | 5508 | 10122 |
| 2003 | 56.92 | 0.20 | 5415 | 10355 |
| 2004 | 56.60 | -0.07 | 6241 | 9168 |
| 2005 | 56.74 | 0.12 | 9500 | 13988 |
| 2006 | 55.19 | -1.09 | 9626 | 15329 |
| 2007 | 56.10 | 2.79 | 9004 | 14177 |
| 2008 | 57.61 | 2.10 | 9500 | 13031 |
| 2009 | 56.22 | -0.41 | 9781 | 9762 |
| 2010 | 56.27 | -4.64 | 9688 | 11271 |
| 2011 | 56.13 | -1.57 | 10380 | 11967 |
| 2012 | 57.54 | 3.17 | 10101 | 9272 |
| 2013 | 57.66 | -1.97 | 9063 | 8124 |
| 2014 | 58.13 | 3.10 | 9459 | 5824 |
| 2015 | 58.33 | 3.56 | 7221 | 7702 |
| 2016 | 59.04 | 0.98 | 7778 | 6765 |
| 2017 | 58.88 | 1.47 | 5430 | 6295 |
