# Supplementary material for: It’s not all abundance: Detectability and accessibility of food also explain breeding investment in long-lived marine animals
Source: PLoS One. 2022 Sep 21;17(9):e0273615. doi: 10.1371/journal.pone.0273615 (PMC9491606; doi:10.1371/journal.pone.0273615)
Supplement: S12 Table — (DOCX) [file pone.0273615.s012.docx]

S12 Table. Estimates ± Standard Error of the best explanatory model (Model 1 in Tables 1 and S1) for the Scopoli’s shearwater.

| Estimates ± SE | Model 1 |
| --- | --- |
| Intercept | 78.53 ± 1.50 |
| Winter NAO | -0.20 ± 0.07 |
| WaveHeight | -7.19 ± 2.09 |
| DiscardsPC | 9.42e+3 ± 3.59e+3 |
| WaveHeight : DiscardsPC | -1.91e+4 ± 4.92e+3 |
