# Supplementary material for: It’s not all abundance: Detectability and accessibility of food also explain breeding investment in long-lived marine animals
Source: PLoS One. 2022 Sep 21;17(9):e0273615. doi: 10.1371/journal.pone.0273615 (PMC9491606; doi:10.1371/journal.pone.0273615)
Supplement: S13 Table — (DOCX) [file pone.0273615.s013.docx]

S13 Table. Estimates Standard Error for the best explanatory models (Models 1-5 in Tables 2 and S2) for the Sandwich tern

| Estimates ± SE | Model 1 | Model 2 | Model 3 | Model 4 | Model 5 |
| --- | --- | --- | --- | --- | --- |
| Intercept | 37,48 ± 1.21 | 35.87 ± 0.61 | 37.80 ± 1.24 | 35.96 ± 0.62 | 38.44 ± 4.07 |
| Winter NAO | -0,21 ± 0.07 | -0.14 ± 0.05 | -0.23 ± 0.07 | -0.15 ± 0.06 | -0.20 ± 0.07 |
| Wind3Q | -0,01 ± 0.00 | -0.01 ± 0.00 | -0.01 ± 0.00 | -0.01 ± 0.00 | -0.01 ± 0.02 |
| Turbidity | -17,20 ± 11.17 |  | -19.3 ± 11.3 |  | -29.88 ± 52.42 |
| Compet. By YLG |  |  | -0.00 ± 0.00 | -0.00 ± 0.00 |  |
| Wind3Q : Turbidity |  |  |  |  | 0.07 ± 0.29 |
