# Supplementary material for: It’s not all abundance: Detectability and accessibility of food also explain breeding investment in long-lived marine animals
Source: PLoS One. 2022 Sep 21;17(9):e0273615. doi: 10.1371/journal.pone.0273615 (PMC9491606; doi:10.1371/journal.pone.0273615)
Supplement: S14 Table — (DOCX) [file pone.0273615.s014.docx]

S14 Table. Estimates Standard Error for the best explanatory model (Model 1 in Tables 3 and S3) for the Audouin’s gull.

| Estimates ± SE | Model 1 |
| --- | --- |
| Intercept | 63.59 ± 1.08 |
| Winter NAO | 0.15 ± 0.02 |
| Intrasp. Compet. | -6.22e-4 ± 1.11e-4 |
| Compet. by YLG | -4.81e-4 ± 1.22e-4 |
| Intrasp. Compet. : Compet. by YLG | 4.41e-8 ± 1.22e-8 |
