# Supplementary material for: It’s not all abundance: Detectability and accessibility of food also explain breeding investment in long-lived marine animals
Source: PLoS One. 2022 Sep 21;17(9):e0273615. doi: 10.1371/journal.pone.0273615 (PMC9491606; doi:10.1371/journal.pone.0273615)
Supplement: S3 Fig — (DOCX) [file pone.0273615.s017.docx]

S17 Figure. Mean egg volume observed v.s. the mean egg volume predicted by the best explanatory model (Model 1 in Tables 3 and S3) for the Audouin’s Gull.
