# Supplementary material for: It’s not all abundance: Detectability and accessibility of food also explain breeding investment in long-lived marine animals
Source: PLoS One. 2022 Sep 21;17(9):e0273615. doi: 10.1371/journal.pone.0273615 (PMC9491606; doi:10.1371/journal.pone.0273615)
Supplement: S1 File — (DOCX) [file pone.0273615.s018.docx]

**Supporting Information References**

Bécares, J. et al., (2015). Modelling Terrestrial and Marine Foraging Habitats in Breeding Audouin’s Gulls Larus audouini: Timing Maters. Plos One 10(4): e0120799. pmid:25875597.

Fasola, M. et al., (1990). Foraging Ranges of an Assemblage of Mediterranean Seabirds. Colonial Waterbirds, 13:1, 72-74.

Reyes-González et al 2017. Migración y Ecología Espacial de las Poblaciones Españolas de Pardela Cenicienta. Monografía nº3 del programa Migra. SEO/BirdLife. Madrid. https://doi.org/10.31170/0056.
