# Supplementary material for: It’s not all abundance: Detectability and accessibility of food also explain breeding investment in long-lived marine animals
Source: PLoS One. 2022 Sep 21;17(9):e0273615. doi: 10.1371/journal.pone.0273615 (PMC9491606; doi:10.1371/journal.pone.0273615)

Contents

Load data **1**

Multiple regression **3**

Without scaling 3

R-squared 3

With scaling 4

Generalized Linear Model **5**

Diagnostic Plots **6**

Variance Inflation Factor 6

Added-Variable plot 7

Predictions Plot **8**

Relative importance **9**

Load data

```
library(tidyverse)
sw <- read.table("DiomedeaUltim_II.txt", header = T)
#no readme found

op <- par(mfrow=c(2,2))
hist(sw$Volume)
hist(sw$W_NAO)
hist(sw$WaveHeight)
hist(sw$DiscardsPC)
```

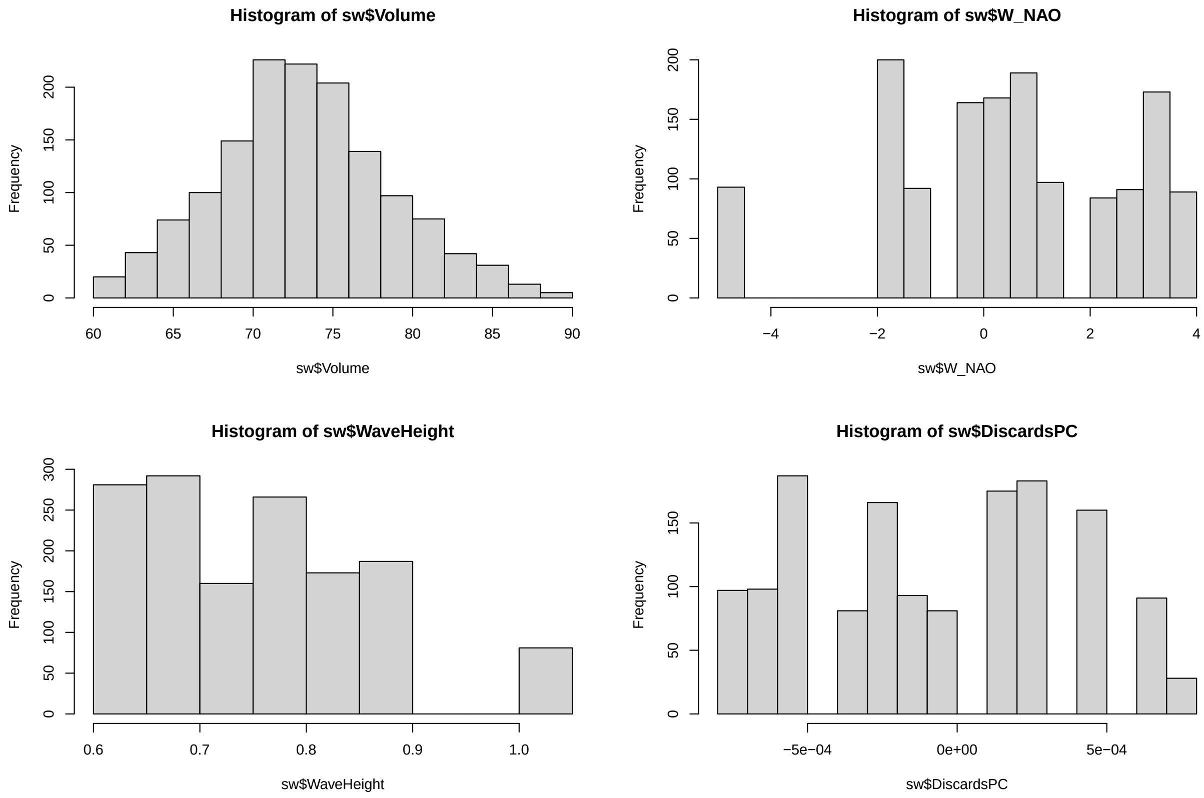


```
par(op)

#standardization
sw_s <- sw %>%
  mutate(WaveHeight_s=scale(WaveHeight)[,1],
         DiscardsPC_s=scale(DiscardsPC)[,1],
         W_NAO_s=scale(W_NAO)[,1])
```

Multiple regression

Without scaling

```
m_best <- lm(Volume ~ W_NAO + WaveHeight + DiscardsPC + WaveHeight:DiscardsPC, data=sw)
summary(m_best)#outputs that correspond to Table S1
```

```
##
## Call:
## lm(formula = Volume ~ W_NAO + WaveHeight + DiscardsPC + WaveHeight:DiscardsPC,
##     data = sw)
##
## Residuals:
##      Min       1Q   Median       3Q      Max
## -14.2522  -3.4077  -0.2381   3.1022  16.3188
##
## Coefficients:
##                        Estimate Std. Error t value Pr(>|t|)
## (Intercept)            7.853e+01  1.507e+00  52.119  < 2e-16 ***
## W_NAO                 -2.060e-01  7.187e-02  -2.867  0.00421 **
## WaveHeight            -7.191e+00  2.094e+00  -3.435  0.00061 ***
## DiscardsPC             9.426e+03  3.597e+03   2.621  0.00887 **
## WaveHeight:DiscardsPC -1.191e+04  4.920e+03  -2.421  0.01559 *
## ---
## Signif. codes:  0 '***' 0.001 '**' 0.01 '*' 0.05 '.' 0.1 ' ' 1
##
## Residual standard error: 5.328 on 1435 degrees of freedom
## Multiple R-squared:  0.03275,    Adjusted R-squared:  0.03005
## F-statistic: 12.15 on 4 and 1435 DF,  p-value: 1.033e-09
```

R-squared

```
summary(m_best)$r.squared #very low
```

```
## [1] 0.0327465
```

With scaling

```r
m_best_s <- lm(Volume ~ W_NAO_s + WaveHeight + DiscardsPC + WaveHeight:DiscardsPC, data=sw_s)
summary(m_best_s)
```

```
##
## Call:
## lm(formula = Volume ~ W_NAO_s + WaveHeight + DiscardsPC + WaveHeight:DiscardsPC,
##     data = sw_s)
##
## Residuals:
##      Min       1Q   Median       3Q      Max
## -14.2522  -3.4077  -0.2381   3.1022  16.3188
##
## Coefficients:
##                         Estimate Std. Error t value Pr(>|t|)
## (Intercept)            7.843e+01  1.519e+00  51.621  < 2e-16 ***
## W_NAO_s               -4.443e-01  1.550e-01  -2.867  0.00421 **
## WaveHeight            -7.191e+00  2.094e+00  -3.435  0.00061 ***
## DiscardsPC             9.426e+03  3.597e+03   2.621  0.00887 **
## WaveHeight:DiscardsPC -1.191e+04  4.920e+03  -2.421  0.01559 *
## ---
## Signif. codes:  0 '***' 0.001 '**' 0.01 '*' 0.05 '.' 0.1 ' ' 1
##
## Residual standard error: 5.328 on 1435 degrees of freedom
## Multiple R-squared:  0.03275,    Adjusted R-squared:  0.03005
## F-statistic: 12.15 on 4 and 1435 DF,  p-value: 1.033e-09
```

Generalized Linear Model

```r
m_glm <- glm(Volume ~ W_NAO_s + WaveHeight + DiscardsPC + WaveHeight:DiscardsPC,
             family=gaussian(link="identity"), data=sw_s)
summary(m_glm)#exact same outputs as m_best_s
```

```
##
## Call:
## glm(formula = Volume ~ W_NAO_s + WaveHeight + DiscardsPC + WaveHeight:DiscardsPC,
##     family = gaussian(link = "identity"), data = sw_s)
##
## Deviance Residuals:
##      Min        1Q    Median        3Q       Max
## -14.2522   -3.4077   -0.2381    3.1022   16.3188
##
## Coefficients:
##                        Estimate Std. Error t value Pr(>|t|)
## (Intercept)           7.843e+01  1.519e+00  51.621  < 2e-16 ***
## W_NAO_s              -4.443e-01  1.550e-01  -2.867  0.00421 **
## WaveHeight           -7.191e+00  2.094e+00  -3.435  0.00061 ***
## DiscardsPC            9.426e+03  3.597e+03   2.621  0.00887 **
## WaveHeight:DiscardsPC -1.191e+04  4.920e+03  -2.421  0.01559 *
## ---
## Signif. codes:  0 '***' 0.001 '**' 0.01 '*' 0.05 '.' 0.1 ' ' 1
##
## (Dispersion parameter for gaussian family taken to be 28.38574)
##
##     Null deviance: 42113  on 1439  degrees of freedom
## Residual deviance: 40734  on 1435  degrees of freedom
## AIC: 8911.6
##
## Number of Fisher Scoring iterations: 2
```

Diagnostic Plots

http://www.sthda.com/english/articles/39-regression-model-diagnostics/161-linear-regression-assumptions-and-diagnostics-in-r-essentials/

```r
op <- par(mfrow = c(2, 2)) ; plot(m_best, pch = 16, col = '#006EA1'); par(op)
```

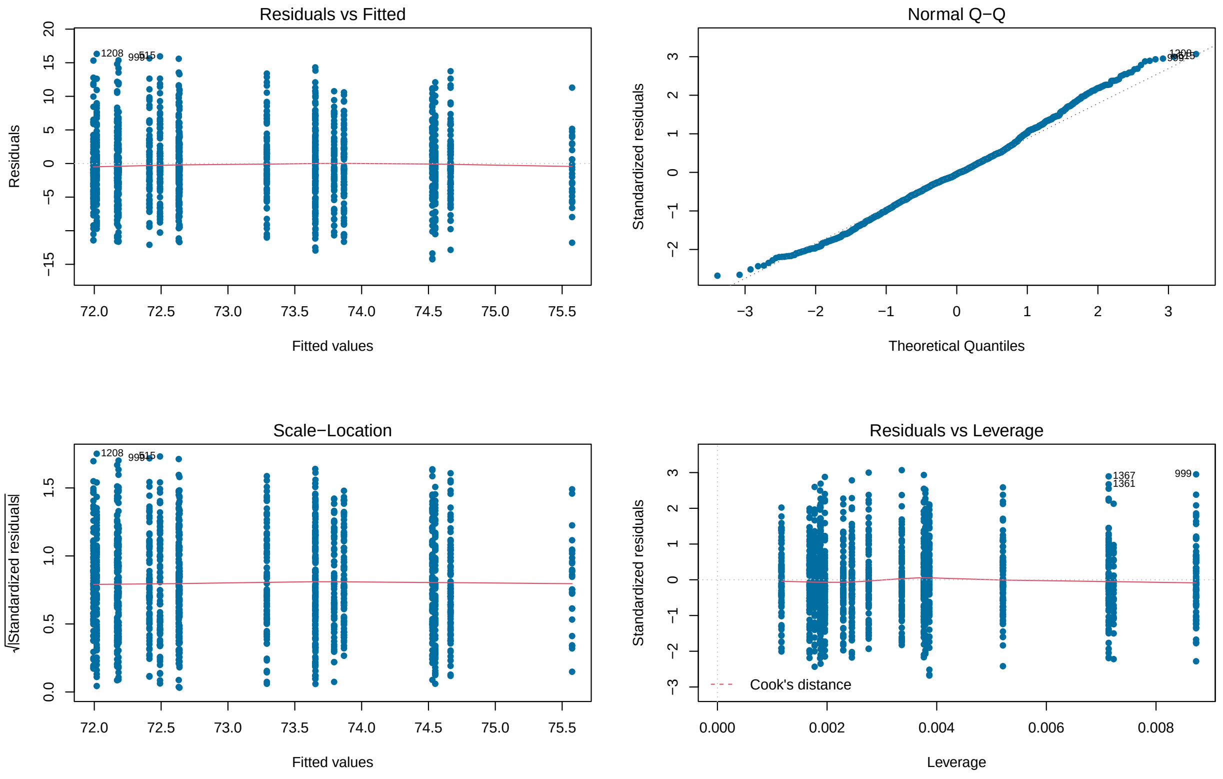

Variance Inflation Factor

```r
library(car)
vif(m_best)
```

```
##                  W_NAO            WaveHeight            DiscardsPC
##               1.217680              2.439105            129.332130
## WaveHeight:DiscardsPC
##             137.125941
```

Added-Variable plot

https://www.statology.org/added-variable-plot-r/

```
library(car)
avPlots(m_best)
```

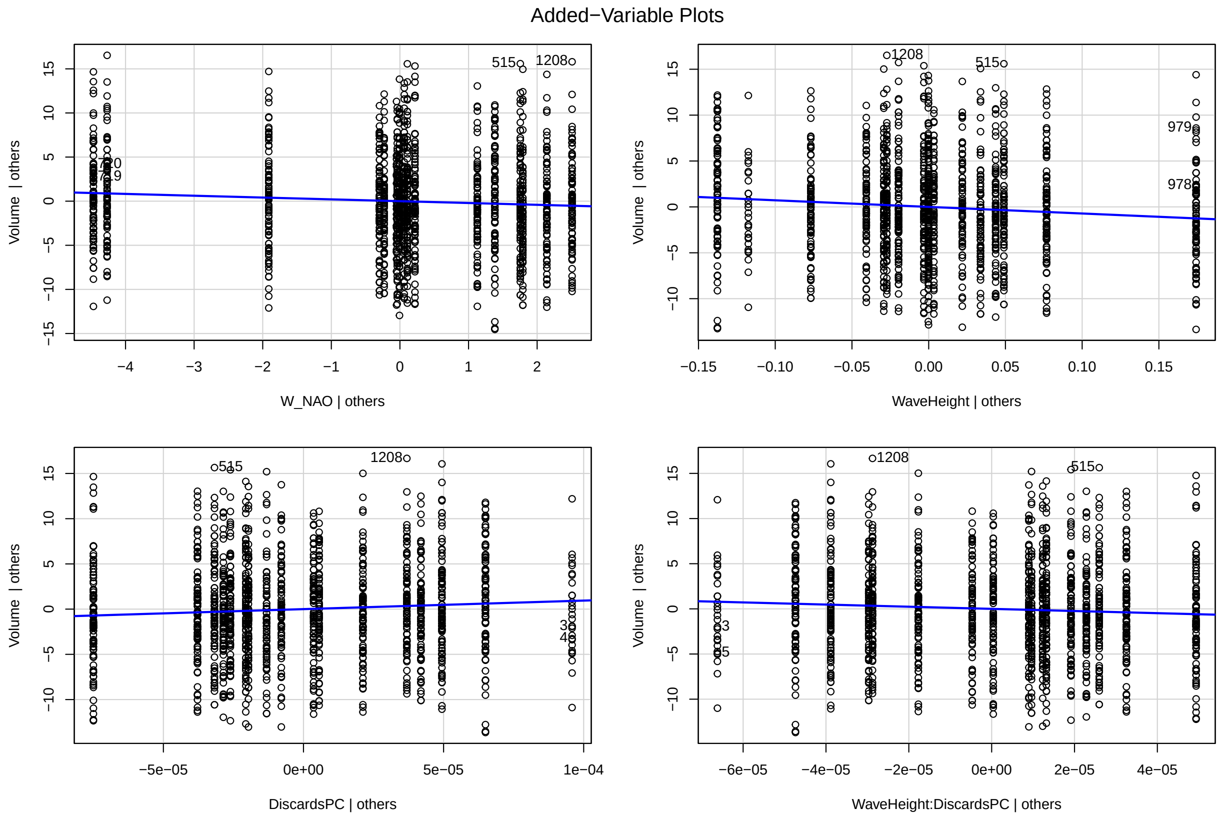

Predictions Plot

https://strengejacke.github.io/ggeffects/articles/ggeffects.html

```
library(ggeffects)
mydf <- ggpredict(m_best,
                  terms = c("W_NAO",
                            "WaveHeight [0.6,0.7,0.8,0.9,1.0]",
                            "DiscardsPC [-8e-4,-5e-4,-1e-4,3e-4,8e-4]"))

ggplot(mydf, aes(x, predicted, colour = group)) +
  facet_grid(~facet)+
  geom_line()+
  labs(
    x = get_x_title(mydf),
    y = get_y_title(mydf),
    colour = get_legend_title(mydf)
  )+
  theme_bw(base_size = 22)+
  theme(plot.title=element_text(hjust=.5))+
  ggtitle("DiscardsPC")
```

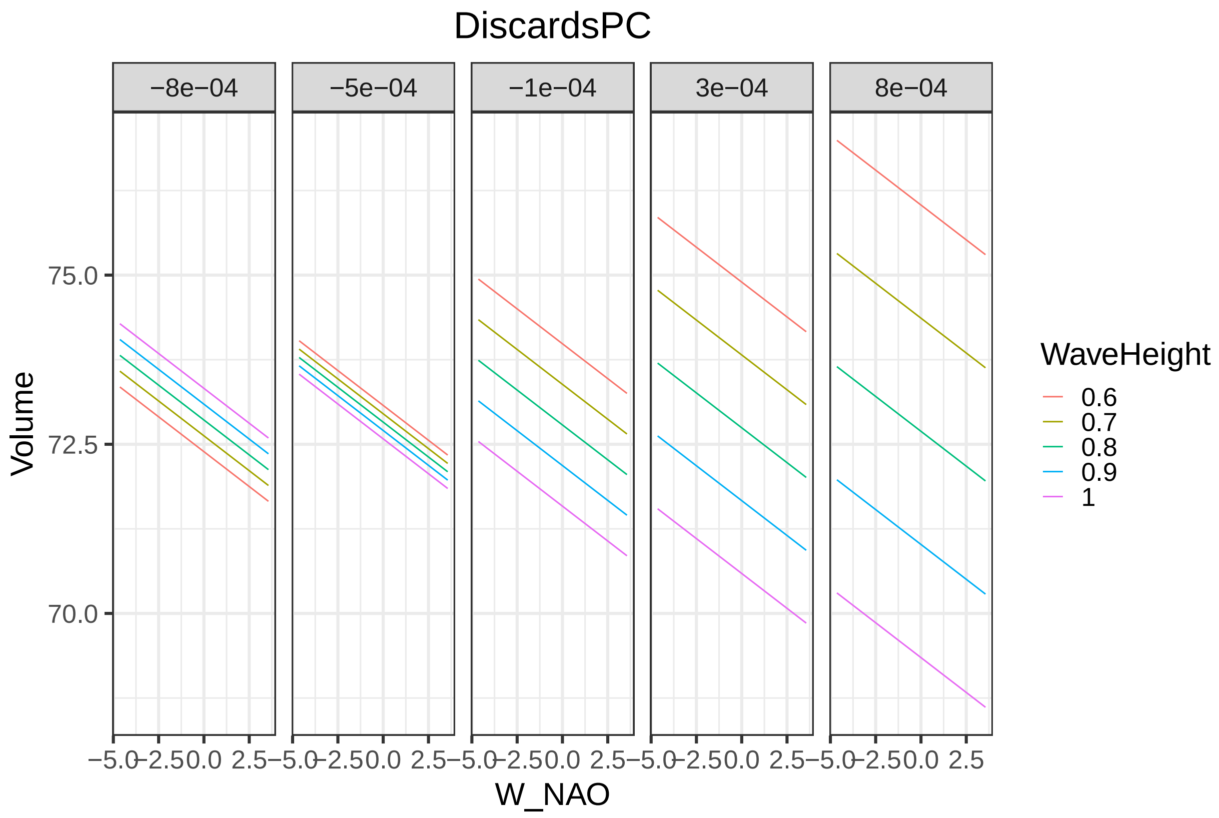

Relative importance

```
anova(m_best)
```

```
## Analysis of Variance Table
##
## Response: Volume
##                        Df Sum Sq Mean Sq F value    Pr(>F)
## W_NAO                   1    781  781.19 27.5206 1.787e-07 ***
## WaveHeight              1    289  288.88 10.1771  0.001453 **
## DiscardsPC              1    143  142.55  5.0218  0.025182 *
## WaveHeight:DiscardsPC   1    166  166.41  5.8626  0.015589 *
## Residuals            1435  40734   28.39
## ---
## Signif. codes:  0 '***' 0.001 '**' 0.01 '*' 0.05 '.' 0.1 ' ' 1
```

```
library(rwa)
sw_s %>%
  rwa(outcome = "Volume",
      predictors = c("W_NAO_s","WaveHeight_s", "DiscardsPC_s"),
      applysigns = F) %>%
  plot_rwa()+
  theme_bw(base_size = 22)
```

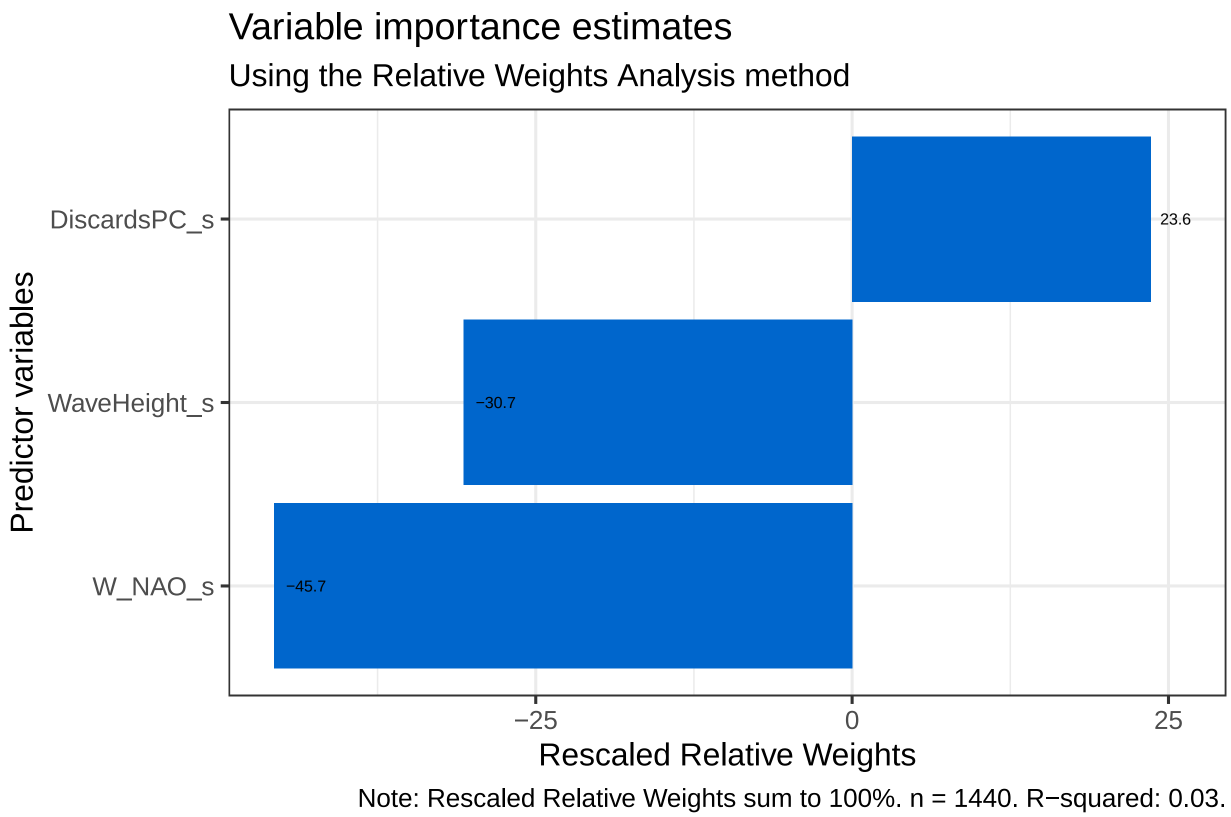

Supplement: Attachment — Submitted filename: sheawaters.pdf [file pone.0273615.s020.pdf]
