# Supplementary material for: It’s not all abundance: Detectability and accessibility of food also explain breeding investment in long-lived marine animals
Source: PLoS One. 2022 Sep 21;17(9):e0273615. doi: 10.1371/journal.pone.0273615 (PMC9491606; doi:10.1371/journal.pone.0273615)
Supplement: Attachment — Submitted filename: Rebuttal letter.docx [file pone.0273615.s019.docx]

**Rebuttal Letter**

Dear editor and reviewers, on behalf of all authors, thank you very much for accepting reviewing our manuscript.

- I have found a little mistake regarding to my email address: “enric.real@ieo.es” is not my email address. It is true that I sign my scientific manuscripts as “Enric Real” but my email address is “[enrique.real@ieo.es](mailto:enrique.real@ieo.es)”. Please change this email address for future emails.

If you would like to make changes to your financial disclosure, please include your updated statement in your cover letter.

- Thank you very much. We have included a new statement in this regard in the cover letter.

1. Please ensure that your manuscript meets PLOS ONE's style requirements, including those for file naming. The PLOS ONE style templates can be found at

<https://journals.plos.org/plosone/s/file?id=wjVg/PLOSOne_formatting_sample_main_body.pdf> and

https://journals.plos.org/plosone/s/file?id=ba62/PLOSOne_formatting_sample_title_authors_affiliations.pdf

- Thank you. In this new version the manuscript meets all PLOS ONE’s style requirements.

2. In your Methods section, please provide additional information regarding the permits you obtained for the work. Please ensure you have included the full name of the authority that approved the field site access and, if no permits were required, a brief statement explaining why.

- Thank you for this comment. We have added this information in the Methods section.

3. Please amend either the title on the online submission form (via Edit Submission) or the title in the manuscript so that they are identical.

- We apologize for this mistake. The title of the online submission and the manuscript are now identical.

4. Thank you for stating the following in the Acknowledgments Section of your manuscript:

“We are grateful to everyone who helped with fieldwork and provided data, images, and logistic support, especially the staff of the Ebro Delta Natural Park. Funds were supplied by grants CGL2013-42203-R and CGL2017-85210-P (MCIU/AEI/FEDER, UE). The study also received funding from the European Commission’s Horizon 2020 Research and Innovation Program under Grant Agreement no. 634495 for the project Sci¬ence, Technology, and Society Initiative to Minimize Unwanted Catches in European Fisheries (MINOUW). MG was partially funded by Govern Balear. ASA was supported by a Ramón y Cajal contract (RYC-2017- 22796).”

We note that you have provided additional information within the Acknowledgements Section. Please note that funding information should not appear in the Acknowledgments section or other areas of your manuscript. We will only publish funding information present in the Funding Statement section of the online submission form.

- Yes, we understand and we have removed the funding information from the manuscript.

Please remove any funding-related text from the manuscript and let us know how you would like to update your Funding Statement. Currently, your Funding Statement reads as follows:

“Funds were supplied by grants CGL2013-42203-R and CGL2017-85210-P (MCIU/AEI/FEDER, UE) respectively. The study also received funding from the European Commission’s Horizon 2020 Research and Innovation Program under Grant Agreement no. 634495 for the project Sci-ence, Technology, and Society Initiative to Minimize Unwanted Catches in European Fisheries (MINOUW). MG was partially funded by Govern Balear. ASA was supported by a Ramón y Cajal contract (RYC-2017- 22796). ”

- Thank you very much for this change. We agree this paragraph is correct as Funding Statement. We have added the marked sentence at the end of the paragraph as follows:

“Funds were supplied by grants CGL2013-42203-R and CGL2017-85210-P (MCIU/AEI/FEDER, UE) respectively. The study also received funding from the European Commission’s Horizon 2020 Research and Innovation Program under Grant Agreement no. 634495 for the project Sci-ence, Technology, and Society Initiative to Minimize Unwanted Catches in European Fisheries (MINOUW). MG was partially funded by Govern Balear. ASA was supported by a Ramón y Cajal contract (RYC-2017- 22796). The funders had no role in study design, data collection and analysis, decision to publish, or preparation of the manuscript.”

Please include your amended statements within your cover letter; we will change the online submission form on your behalf.

- Thanks, we have included the new funding statement within the cover letter.

5. Thank you for stating the following financial disclosure:

 “Funds were supplied by grants CGL2013-42203-R and CGL2017-85210-P (MCIU/AEI/FEDER, UE) respectively. The study also received funding from the European Commission’s Horizon 2020 Research and Innovation Program under Grant Agreement no. 634495 for the project Sci-ence, Technology, and Society Initiative to Minimize Unwanted Catches in European Fisheries (MINOUW). MG was partially funded by Govern Balear. ASA was supported by a Ramón y Cajal contract (RYC-2017- 22796).”

Please state what role the funders took in the study.  If the funders had no role, please state: "The funders had no role in study design, data collection and analysis, decision to publish, or preparation of the manuscript."

- *Thanks, we have stated that funders had no role in the study.*

“Funds were supplied by grants CGL2013-42203-R and CGL2017-85210-P (MCIU/AEI/FEDER, UE) respectively. The study also received funding from the European Commission’s Horizon 2020 Research and Innovation Program under Grant Agreement no. 634495 for the project Sci-ence, Technology, and Society Initiative to Minimize Unwanted Catches in European Fisheries (MINOUW). MG was partially funded by Govern Balear. ASA was supported by a Ramón y Cajal contract (RYC-2017- 22796). The funders had no role in study design, data collection and analysis, decision to publish, or preparation of the manuscript.”

If this statement is not correct you must amend it as needed.

Please include this amended Role of Funder statement in your cover letter; we will change the online submission form on your behalf.

6. In your Data Availability statement, you have not specified where the minimal data set underlying the results described in your manuscript can be found. PLOS defines a study's minimal data set as the underlying data used to reach the conclusions drawn in the manuscript and any additional data required to replicate the reported study findings in their entirety. All PLOS journals require that the minimal data set be made fully available. For more information about our data policy, please see <http://journals.plos.org/plosone/s/data-availability>.

Upon re-submitting your revised manuscript, please upload your study’s minimal underlying data set as either Supporting Information files or to a stable, public repository and include the relevant URLs, DOIs, or accession numbers within your revised cover letter. For a list of acceptable repositories, please see <http://journals.plos.org/plosone/s/data-availability#loc-recommended-repositories>.  Any potentially identifying patient information must be fully anonymized.

- Yes, we have already uploaded this data to a public repository and the link to access the data has been incorporated within the cover letter.

Important: If there are ethical or legal restrictions to sharing your data publicly, please explain these restrictions in detail. Please see our guidelines for more information on what we consider unacceptable restrictions to publicly sharing data: <http://journals.plos.org/plosone/s/data-availability#loc-unacceptable-data-access-restrictions>. Note that it is not acceptable for the authors to be the sole named individuals responsible for ensuring data access.

We will update your Data Availability statement to reflect the information you provide in your cover letter.

7. We note that you have stated that you will provide repository information for your data at acceptance. Should your manuscript be accepted for publication, we will hold it until you provide the relevant accession numbers or DOIs necessary to access your data. If you wish to make changes to your Data Availability statement, please describe these changes in your cover letter and we will update your Data Availability statement to reflect the information you provide.

- This point is not applicable now as we have already uploaded this data to a public repository and the link to access the data has been incorporated within the cover letter.

8. Please include captions for your Supporting Information files at the end of your manuscript, and update any in-text citations to match accordingly. Please see our Supporting Information guidelines for more information: <http://journals.plos.org/plosone/s/supporting-information>.

- Thanks, captions for the Supporting information have been included at the end of the manuscript.

[Note: HTML markup is below. Please do not edit.]

Reviewers' comments:

Reviewer's Responses to Questions

**Comments to the Author**

1. Is the manuscript technically sound, and do the data support the conclusions?

The manuscript must describe a technically sound piece of scientific research with data that supports the conclusions. Experiments must have been conducted rigorously, with appropriate controls, replication, and sample sizes. The conclusions must be drawn appropriately based on the data presented.

Reviewer #1: Yes

Reviewer #2: Yes

Reviewer #3: Partly

2. Has the statistical analysis been performed appropriately and rigorously?

Reviewer #1: Yes

Reviewer #2: Yes

Reviewer #3: No

3. Have the authors made all data underlying the findings in their manuscript fully available?

The [PLOS Data policy](http://www.plosone.org/static/policies.action#sharing) requires authors to make all data underlying the findings described in their manuscript fully available without restriction, with rare exception (please refer to the Data Availability Statement in the manuscript PDF file). The data should be provided as part of the manuscript or its supporting information, or deposited to a public repository. For example, in addition to summary statistics, the data points behind means, medians and variance measures should be available. If there are restrictions on publicly sharing data—e.g. participant privacy or use of data from a third party—those must be specified.

Reviewer #1: No

Reviewer #2: Yes

Reviewer #3: Yes

4. Is the manuscript presented in an intelligible fashion and written in standard English?

PLOS ONE does not copyedit accepted manuscripts, so the language in submitted articles must be clear, correct, and unambiguous. Any typographical or grammatical errors should be corrected at revision, so please note any specific errors here.

Reviewer #1: Yes

Reviewer #2: Yes

Reviewer #3: Yes

 5. Review Comments to the Author

Please use the space provided to explain your answers to the questions above. You may also include additional comments for the author, including concerns about dual publication, research ethics, or publication ethics. (Please upload your review as an attachment if it exceeds 20,000 characters)

Reviewer #1: This paper correlates long-term monitoring data on egg volume in three model marine bird species and values of the NAO Index as well as local-scale food related variables. The manuscript is well written and provides an interesting contribution to this topic. I have several comments to further improve clarity of the paper.

Keywords should be listed alphabetically.

- Thank you, we have modified this and keywords are now listed alphabetically

The Introduction section would benefit from adding some hypotheses and/or predictions.

- Yes, our hypotheses is in the last sentence of the Introduction section “*Given the smaller area used by seabirds during the breeding period relative to the winter distribution, we expect local variables to be a better predictor than the North Atlantic Oscillation index on egg volume”*.

L 99 and elsewhere: Please avoid ") (".

- Thank you, this has been already corrected.

L 176: Please introduce and justify the use of interactions.

- Totally agree, we have added a sentence which introduce and justify the evaluation of potential statistical interactions between covariates.

L 178: Please remove dash in "R- Development team 2014".

- Thank you, dash in "R- Development team 2014" has been removed.

L 179: Or simply by 1000?

- We agree that this aids in better readability, so it has been modified as well.

L 191: The study species is in singular, but in L 205 and 219 plural.

- Thank you, now they are all in singular.

L 220-2: Please specify the directions of the significant relationships.

- Yes, this information had been specified for the Scopoli’s shearwaters only. Now we have incorporated this information not only for Audouin gulls, but also for Sandwich terns.

L 271-3: Not fully clear to me.

- Yes, the sentence was not much clear. Now we have simplified this sentence for better understanding.

L 299: Here I would write about conditions rather than variables.

- Thank you, we have changed variables by conditions.

L 585: Please better explain wind quartiles or refer to the methods section (see also supplement).

- Thank you. Now we have added “(see methods section)” in Tables 1,2, S1, S2 and S3.

Fig. 3: Please improve figure resolution.

- Figures were submitted in TIF format and high resolution. However figures have no high resolution in the final draft in pdf.

Supplement: Please use decimal points and the same number of decimals in tables.

- Thank you, now we have used decimal points in the Supplementary material and al the tables have the same number of decimals.

Table S7: P-values cannot be higher than 1. Do the authors mean <0.05?

- Sorry for this mistake, as you say what we mean is p value <0.05. We have corrected in Tables S6. S7 and S8.

Reviewer #2: It’s not all availability: detectability and accessibility to food also explain breeding investment in long-lived animals

General comments
This paper addresses some important and largely ignored issues in ecology, specifically the relative roles of large-scale and local-scale drivers, and the metrics used to explain (eg food abundance vs per capita abundance), demographic response variables for long-lived species. The study used a comprehensive data set from three species with differing life history strategies. I found the paper to be well-written, concise and with a clearly defined analysis and message. I have no major comments or criticisms, but list some minor comments below. I recommend the paper publication is suitable for publication in PLOS ONE

Specific comments
Line 73.’ Bet-hedgers species’ should be ‘bet-hedger species’ or ‘bet-hedging species’?

- Thank you, we have changed “bet-hedger species” by “bet-hedgers”.

Line 74. I’m not sure what you mean by ‘slower’ life history. Can you expand a little and/or contrast more explicitly with a bet-hedging strategy?

- We agree this sentence was not clear enough and maybe unnacurate. Mow we have simplified this sentence as follows: “*Furthermore, terns and gulls can modulate clutch size depending on environmental conditions, whereas shearwaters lay a single egg.”*

Lines 89-91. You mention two issues (food detectability and availability) and cite three related processes (sea conditions, per capita food abundance and competition) without making clear their associations. Presumably sea conditions relates to detectability and per capita abundance and competition to availability? I think it would help the reader to make the explicit association here between the issues and processes.

- The issues we mention here are detectability and accessibility of food but not its availability. Sea conditions influence both, the detectability of food (eg turbidity, waves…) and its accessibility. For instance, in high swell conditions, natural prey are found at greater depths being less accessible. Competition also influence food accessibility. For example, intra- and –interspecific competition during the discard process make discards less accessible. However we agree this sentence should be improved. We have changed this sentence as follows: “*For these local-scale variables, we included the factors influencing food detectability and accessibility (foraging conditions and both intraspecific and interspecific competition) and per capita food abundance.*”

Lines 102-103. In reading this description of the common foraging area I was expecting to see a semi-circle of 100 km radius centred on the Ebro delta in Fig 1, but the green delineation seems to be indicated by a combination of the shelf area 100 km to either side of the delta along the coast. You could perhaps fine-tune the description a bit.

- Thank you, we have also changed this sentence as follows: “*thus, we considered the continental shelf of a marine area of 100km radius centred on the Ebro Delta as representative of their common foraging area*”

Lines 131-132. The issues of detectability and availability are central to the paper, but its not clear to me (yet) how they differ in principle. For example, if it is hard to detect food because the water is turbid, then it could be argued that the food is also inaccessible even if it is abundant, because it is unseen by whatever process. I am most familiar with these terms in the abundance estimation literature, where say in an aerial survey of a marine species, availability refers to whether an animal is near the surface and therefore potentially detectable, and detectability refers to the chance of seeing it given it is near. Can you define these terms in the context of your paper and the three species which have differing foraging strategies?

- Thanks for this observation. We have added the following sentence in the Methods section to clarify this: “*We assume that in the foraging process, animals first detect food and then try to access it.* *Therefore, we use the term accessibility to refer to those physical barriers making the food item difficult to reach once it is detected.”*

At the end of the Introduction section, in this sentence “*For these local-scale variables, we included the factors potentially influencing food detectability and accessibility”*, we have added the term “potentially”, because we hipothesize that these factors may influence detectability and accessibility of food and ultimately egg volume.

Regarding the relationship between these terms (detectability and accessibility) and species-specific feeding strategies, we have improved the explanations of feasible mechanisms in the Discussion section, specifying whether each covariate considered affects detectability, accessibility, or both, especially in those sentences where it had not been made clear enough..

I see on lines 270-271 the example of prey being at greater depths is seen to influence both detectability and availability as if they are the same thing? Is this the intent?

- No, this was not the intention. What we are referring to here is that when prey are located deeper: i) they will be more difficult to detect from the air and ii) they will be less accessible because deeper and longer dives will be required by seabirds. This is also well illustrated in [39 and 63].

Line 173. Insert ‘by’ between calculated and divided: ie ‘calculated by dividing’

- Thanks. It has been already inserted.

Lines 183-184. There is an imbalance of parentheses here

- Thanks, this has been already corrected too.

Lines 213-214. Interspecific competition of sandwich terns with yellow legged gulls…is this consistent with their dive depths (terns are short diving plungers)

- We consider that competition could occur in at least two different ways here. Firstly, when both species compete for discards and secondly, due to the kleptoparasitism exerted by yellow-legged gulls on terns, which probably affects both discards and natural prey.

Lines 282-283. I am interested in whether the finding of inter-specific competition between Audouins gull and the yellow legged gull is related to them having similar foraging strategies in relation to depth. You say early in the paper that that gulls are surface feeders, and if this applied to both these species, you would expect a higher chance of interspecific competition between these two species than with the other species, and this could be useful to have in the discussion.

- Yes, competition between these two species has been well documented by several authors, especially for fishery discards. We have modified a sentence at the end of the Discussion section which includes some references in this regard. Some studies have also shown that in the western Mediterranean, yellow-legged gulls and Audouin's gulls compete for discards in the vicinity of fishing boats. In contrast, shearwaters (much better divers) often position themselves 30-50m to the rear of boats to reach discards that have sunk deeper.

Reviewer #3: The authors present an analysis of annual egg-volume in three seabird species. Overall, I think there is merit in this analysis, but the authors need to give much more detail in their methods and results. The author frame the work in terms of comparing large-scale and local climate variables, but I am not convinced their methods properly compared these two variables. They considered both, but there are no details on statistically evaluating whether one had a smaller or larger impact than the other.

- Thanks for the comment. In one hand, we also include models where covariates were taken one by one to assess their relative importance in terms of AIC. These AICs are found in the supporting information (Tables S1, S2 and S3). On the other hand, we assessed the relative importance of some covariates by calculating their R^2^ (see Data Analysis in the Methods section for more details).

I’d also like to see the methods section written so that another research could use the same dataset and replicate the analysis exactly.

- Yes, now datasets are available as they have been uploaded to a public repository. The links to access to datasets have been also delivered to Plos One. In the methods section there is all necessary information to replicate the analysis exactly.

I am also wary of the conclusions drawn in the discussion of the study. The study uses many proxies of different climate variables to identify statistical correlations. The authors need to be more cautious in their interpretation to not assume they are observing actual effects of these variables on egg-volume. Finally, I’d like to see the estimates from each of the top models identified

- Thank you for the comment. These estimates are now in the supporting information in Tables S12, S13 and S14.

as well as some plots showing the relationship between predictors and egg-volume. I think it is fine if these go in the supplementary, but they are needed to properly evaluate the conclusions drawn in this study.

- Thanks again, these plots are now in the supporting information (Figures S1, S2 and S3).

Line 79-83: I think the wording here is not careful enough. It is the among individual variation that matters more for your argument here. If it is of environmental origin, but has a permanent impact of egg size throughout the lifetime of an adult it will make egg-size just as inflexible in response to environmental conditions. However, heritabilities and repeatabilities are dependent on the environment they are measured in and don’t tell use much about flexibility in different environments– so the measurements in Christians 2002 don’t have much bearing on whether or not egg size will be flexible or plastic in response to environmental conditions. I think you should just remove the first part of the sentence and remark that egg-size is used as an indicator of environmental conditions.

- Several researchers have said that the egg volume is not a good indicator of environmental conditions since this parameter is strongly dependent on females' size. We are fully aware of the importance of female size, which in some birds can account for up to 80% of the total egg volume, but many studies show that the other ~20% is much more flexible and dependent on environmental conditions, including food availability. We consider it is very important to clarify this so, that's why we decided to include this sentence.

Line 83-85: Egg volume can be correlated with chick growth and survival. I think it is important to not suggest that it is always correlated with chick growth and survival

- Thank you. We have already changed this.

Line 86: “Using long-term monitoring of data of these three species...” Clearly name species here. You’ve mentioned them above, but it isn’t clear that they are the focus of your paper.

- Thanks for the comment. We have modified this sentence including the names of each study species.

Missing in the introduction is a brief sentence indicating why the different variables to affect egg volume. Is this through food resources, stressful conditions?

- In Lines 86-94 in the Introduction section we explain that we use different variables potentially affecting food detectability and accessibility and per-capita food abundance (all of which are food-related variables that affect food intake) as predictors of the egg volume. We consider that it can be assumed by the reader that these variables influence egg volume through food intake. However, we can change this paragraph if necessary.

Are egg measurements repeated across females? Do you have identification of the females for each clutch? Observations are likely not independent and you have not accounted for this non-independence. Even if you do not have size measurements it might be important to consider female ID. If this is a smaller subset of the data it would be valuable to see the analysis with female ID or female size and see how these results compare to your overall results.

- We agree that considering this would be valuable but, females data was not available so we assumed to be a random and additive component of the total variance.

Data analysis

Do you only assess linear trends in your analysis?

- Yes, taking into account the number of covariates and spatial scales, we considered that GLMs are an appropriate technique in this case.

How did you decide to include and evaluate interactions?

- When considering possible scenarios in the foraging process. For example, we suspect that when sea conditions are very bad, it is more complicated for seabirds to reach discarded items so we decided to evaluate this interaction.

Line 131-135: You arent really assessing how these variables affect detectability and accessibility of food. You are assuming that these proxies you use are using are related to the detectability and accessibility of food for foraging birds. You make the assumption that a relationship between and egg-volume is because of a relationship with food. Please reword.

- Thank you. We have reworded these paragraph to clarify that these variables are used as proxies of food detectability and accessibility.

Line 133: Table S4 does not seem to have any relationship to this sentence?

- We apologize, this was a mistake. We have removed the words "Table S4" from this part of the manuscript.

You use temporal variance of egg volume throughout, but I think it might be clearer to use among annual variance in egg volume. Temporal could be variance within a breeding season.

- Thanks again, we agree and we have changed ‘temporal variance’ by ‘annual variance’ through the text except on those cases were we specify that it is annually.

In the intra and inter specific competition section please clarify how you evaluated the effects of your population counts on each species. Were the count values included as fixed effects in your models?

- In this study we have not included random effects. Therefore, in order to clarify this we have included a sentence in “Data analysis” section indicating that “*all covariates were taken as fixed effects*.”

Line 180-182: What did you do about collinearity? You need to explain more here.

- Thank you, Only uncorrelated covariates (p value >0.05) were considered together in the models. Now this is also explained in the Data analysis section.

Line 183-184: More information is needed here. How was model selection conducted. How did you conduct model selection? Did you compare all possible model structures? Did you try interactions? Did you use the dredge function in MuMIn? Please explain your process.

- Thank you again. Now we specify that best models were those with the lowest AIC. Regarding to model structures, now we specify this: *“Only model structures making ecological sense according to the species-specific diet and foraging strategies were compared”*. In the first paragraph of Data Analysis section, in the sentence “*GLMs were used to analyze factors affecting the egg volume*” we have also added “*as well as their statistical interactions”*. Dredge function in MuMin was not used for model selection.

Results

It will be important to see the data behind the relationships suggelsted in your top models. I want to see the relationship between egg-volume and and your predictors. For example, what does the egg-volume look like for eggs measured when wave height is high and discards are high vs low wave height and low discards vs low wave height and high discards? You need to report your actual model results somewhere. The top model for each species will be fine, but it is needed to evaluate your conclusions!

- Thanks, we have now added three more tables in the supporting information. These tables show annual values of the observed egg volume and covariates retained by the best explanatory model for each species (See Tables S9, S10 and S11).

Line 216-218: You need to explain what “pretending variables” are.

- Thanks again, we have added a sentence explaining what pretending variables are.

Discussion

Discussion would be clearer if separated by species or by large versus small scale effects. Headings would also be helpful if allowed in journal format.

- Thank you for this observation. In fact, this was our first option for which in addition many hours were dedicated to this purpose. However, when finished, most of authors agreed that the reading was much more difficult to follow and we decided to change it to the current format. We all came to the conclusion that this format works well for other sections of the manuscript such as Methods or Results, but not for the Discussion section. Although we do not recommend it, we can change it if necessary.

Line 232 -236: If for all three species egg- volume was better predicted by including both local and large-scale variables, how can you say the relative importance was different? How did you evaluate this? Is the magnitude of the effect sizes different? If you mean that the effects of the same variable were different among species than you need to say this instead.

- Thanks for the comment. We have changed this sentence accordingly as follows:

“The effects of the wNAO and the local variables measured in spring were different for each study species.”

Line 239-241: Please explain the importance of this effect with respect to the slow life-history of the shearwaters. Why would we expect a carry-over effect in a species with a slow-life history? Especially with respect to reproductive investment. Are shearwaters income or capital breeders?

- We have added the following sentence: “*This result also suggests that Cory's Shearwaters act as capital breeders, however further research is needed in this regard, so this possibility should be taken with caution.*”

Line 258-259: Please explain the logic linking this sentence to the topic of this paragraph?

- Thank you, we have modified this sentence to put it on the context of the paragraph.

Line 263: Is it possible your variables are a poor proxy for food abundance?

- We agree they could be better (e.g. using real amounts of discards), but this type of data was not available for the studied period in the Western Mediterranean. However, the choice of these proxies was not made at random. Considering the high proportion of fish discarded related to the total catch, (especially in the studied period), it makes sense to think that the more trawlers are in the area, the more discards are generated. Regarding natural prey, what we did first was to find out the diet of each species according to scientific literature. Then, from among all prey items we considered those that are most abundant in the study area. Finally, we calculated annual estimates of the abundance of these species. Another potentially important factor is that the study area is a very rich area in terms of prey abundance, suggesting that the abundance of food is not a limiting factor and therefore, detectability and accessibility could be more important.

Line 270: Can you provide better support for this possibility? What are the main prey sources of the shearwaters and is there evidence they are affect by larger waves?

- According to our methodology (see above), for shearwaters, sardines are among the most abundant natural prey in the study area. We have made a second search but we have not found more references supporting the possibility that larger waves may drive small pelagic to deeper waters (apart from Dunn 1973).

Line 277: Reference software error on this line?

- Yes, this error only appears in the draft pdf manuscript. We have changed the reference format so it should be fixed.

Line 291-293: Other possible explanations here?

- There is a very clear density dependence effect on the egg volume of Audouin's gulls because the study area hosts up to 73% of the total world population. Regarding the other two species, perhaps the density dependence has almost no or very little effect on the egg volume.

Conclusions
My main feedback for the conclusions is to change the language so it is less certain. For example replace uses of the word influence with correlated. I think it is important to remember all the results are correlations and do not necessarily concretely prove the conclusions described here.

- Thanks, we have changed the term ‘influence’ by ‘are correlated’

Figure caption three is confusing. Are these predictions from the best models for each species or are these the predictions from models with both spring and winter conditions? Please clarify.

- This figure show; i) the egg volume observed, ii) the egg volume predicted by the best explanatory model (which includes winter and spring conditions), and the wNAO alone. We have eliminated “that consider both, winter- and spring-conditions “ from the figure legend for better understanding.

6. PLOS authors have the option to publish the peer review history of their article ([what does this mean?](https://journals.plos.org/plosone/s/editorial-and-peer-review-process#loc-peer-review-history)). If published, this will include your full peer review and any attached files.


If you choose “no”, your identity will remain anonymous but your review may still be made public.


**Do you want your identity to be public for this peer review?** For information about this choice, including consent withdrawal, please see our [Privacy Policy](https://www.plos.org/privacy-policy).

Reviewer #1: No

Reviewer #2: No

Reviewer #3: No

[NOTE: If reviewer comments were submitted as an attachment file, they will be attached to this email and accessible via the submission site. Please log into your account, locate the manuscript record, and check for the action link "View Attachments". If this link does not appear, there are no attachment files.]

While revising your submission, please upload your figure files to the Preflight Analysis and Conversion Engine (PACE) digital diagnostic tool, <https://pacev2.apexcovantage.com/>. PACE helps ensure that figures meet PLOS requirements. To use PACE, you must first register as a user. Registration is free. Then, login and navigate to the UPLOAD tab, where you will find detailed instructions on how to use the tool. If you encounter any issues or have any questions when using PACE, please email PLOS at [figures@plos.org](mailto:figures@plos.org). Please note that Supporting Information files do not need this step.

*In compliance with data protection regulations, you may request that we remove your personal registration details at any time.* [*(Remove my information/details)*](https://www.editorialmanager.com/pone/login.asp?a=r)*. Please contact the publication office if you have any questions.*
