# Supplementary material for: It’s not all abundance: Detectability and accessibility of food also explain breeding investment in long-lived marine animals
Source: PLoS One. 2022 Sep 21;17(9):e0273615. doi: 10.1371/journal.pone.0273615 (PMC9491606; doi:10.1371/journal.pone.0273615)
Supplement: Attachment — Submitted filename: Response to reviewer 4.docx [file pone.0273615.s021.docx]

PONE-D-21-28485R1

It’s not all abundance: detectability and accessibility to food also explain breeding investment in long-lived animals

PLOS ONE

Dear Dr. Real,

Thank you for submitting your manuscript to PLOS ONE. After careful consideration, we feel that it has merit but does not fully meet PLOS ONE’s publication criteria as it currently stands. Therefore, we invite you to submit a revised version of the manuscript that addresses the points raised during the review process.

Please submit your revised manuscript by Apr 30 2022 11:59PM. If you will need more time than this to complete your revisions, please reply to this message or contact the journal office at [plosone@plos.org](javascript:void(window.open('/imp/dynamic.php?page=compose&to=plosone%40plos.org&popup=1%27,%27%27,%27width=820,height=610,status=1,scrollbars=yes,resizable=yes%27))). When you're ready to submit your revision, log on to https://www.editorialmanager.com/pone/ and select the 'Submissions Needing Revision' folder to locate your manuscript file.

Please include the following items when submitting your revised manuscript:

- A rebuttal letter that responds to each point raised by the academic editor and reviewer(s). You should upload this letter as a separate file labeled 'Response to Reviewers'.
- A marked-up copy of your manuscript that highlights changes made to the original version. You should upload this as a separate file labeled 'Revised Manuscript with Track Changes'.
- An unmarked version of your revised paper without tracked changes. You should upload this as a separate file labeled 'Manuscript'.

If you would like to make changes to your financial disclosure, please include your updated statement in your cover letter. Guidelines for resubmitting your figure files are available below the reviewer comments at the end of this letter.

If applicable, we recommend that you deposit your laboratory protocols in protocols.io to enhance the reproducibility of your results. Protocols.io assigns your protocol its own identifier (DOI) so that it can be cited independently in the future. For instructions see: <https://journals.plos.org/plosone/s/submission-guidelines#loc-laboratory-protocols>. Additionally, PLOS ONE offers an option for publishing peer-reviewed Lab Protocol articles, which describe protocols hosted on protocols.io. Read more information on sharing protocols at <https://plos.org/protocols?utm_medium=editorial-email&utm_source=authorletters&utm_campaign=protocols>.

We look forward to receiving your revised manuscript.

Kind regards,

Vitor Hugo Rodrigues Paiva, Ph.D.
Academic Editor
PLOS ONE

[Note: HTML markup is below. Please do not edit.]

Reviewers' comments:

Reviewer's Responses to Questions

**Comments to the Author**

1. If The authors have adequately addressed your comments raised in a previous round of review and you feel that this manuscript is now acceptable for publication, you may indicate that here to bypass the “Comments to the Author” section, enter your conflict of interest statement in the “Confidential to Editor” section, and submit your "Accept" recommendation.

Reviewer #3: All comments have been addressed

Reviewer #4: (No Response)

2. Is the manuscript technically sound, and do the data support the conclusions?

The manuscript must describe a technically sound piece of scientific research with data that supports the conclusions. Experiments must have been conducted rigorously, with appropriate controls, replication, and sample sizes. The conclusions must be drawn appropriately based on the data presented.

Reviewer #3: Yes

Reviewer #4: No

3. Has the statistical analysis been performed appropriately and rigorously?

Reviewer #3: Yes

Reviewer #4: No

4. Have The authors made all data underlying the findings in their manuscript fully available?

The [PLOS Data policy](http://www.plosone.org/static/policies.action#sharing) requires authors to make all data underlying the findings described in their manuscript fully available without restriction, with rare exception (please refer to the Data Availability Statement in the manuscript PDF file). The data should be provided as part of the manuscript or its supporting information, or deposited to a public repository. For example, in addition to summary statistics, the data points behind means, medians and variance measures should be available. If there are restrictions on publicly sharing data—e.g. participant privacy or use of data from a third party—those must be specified.

Reviewer #3: Yes

Reviewer #4: Yes

5. Is the manuscript presented in an intelligible fashion and written in standard English?

PLOS ONE does not copyedit accepted manuscripts, so the language in submitted articles must be clear, correct, and unambiguous. Any typographical or grammatical errors should be corrected at revision, so please note any specific errors here.

Reviewer #3: Yes

Reviewer #4: Yes

6. Review Comments to the Author

Please use the space provided to explain your answers to the questions above. You may also include additional comments for the author, including concerns about dual publication, research ethics, or publication ethics. (Please upload your review as an attachment if it exceeds 20,000 characters)

Reviewer #3: Thank you for your careful consideration and response to all comments. I appreciate it! I think this paper has been greatly improved and presents some interesting ideas that can be tested for more species!

**The authors:** Thank you for these words and for your constructive comments.

Reviewer #4: This is an interesting study that examines the correlation between egg volume of three different seabird species in relation to proxies of food abundance, accessibility and detectability. However, it is difficult to judge from this manuscript whether these chosen proxies well represent the processes The authors intended to test (see details below).

**The authors:** We thank the reviewer for the comment but that was not the original purpose of our study. The main goal was to assess whether local variables are better predictors than a large-scale climatic index (the North Atlantic Oscillation) of the interannual variations of egg volume (see the Abstract). It is important to highlight that we have not correlated these each covariate with the egg volume. We have built models with a combination of them to predict the interannual variation of the mean egg volume depending on environmental conditions considering different spatial scales.

In addition, the lack of hypotheses and predictions of changes in eggs volume as a function of differences in life history strategies, feeding strategies, and population sizes per species, make the results difficult to follow, and the discussion is for me too focused on the studied species, without really drawing conclusions in a broader context

**The authors:** Many studies assume that the correlation between large-scale indexes and ecological processes is due to the effect of local variables. However, the relative role of these two type of covariates (large-scale indexes vs local conditions) has seldom been investigated. Our starting hypothesis was that local variables are better predictors of the mean egg volume variability than large-scale climatic indices as often assumed. We wrote that “*Given the smaller area used by seabirds during the breeding period relative to the winter distribution, we expect local variables to be a better predictor than the North Atlantic Oscillation index on egg volume”* to clarify this point (see L85-87).

In addition, I am concerned about the way the analyses were conducted and at least presented. This is surely also due to the fact that the available data do not contain any description (e.g. with a readme.txt file) and that no R codes were associated with them.

**The authors:** We agree. All statistical procedures are described in the Methods section but we are happy to share our R-scripts if necessary. We have now added three scripts (one per species) to run all GLM.

So I attached with this review an R code in which I tried to reproduce the best model for the shearwater dataset.

**The authors:** Thank you for this. We appreciate it. We have added the scripts and they are now available for review.

It seems to me that instead of using GLMs, The authors actually simply performed a multiple linear regression (see detailed comments),

**The authors:** This is in part correct multiple regressions are a particular case of linear models in wich error structure is considered normally distributed. The main difference is that parameters in multiple regressions are estimated using least of squares methods while here we used likelihood based procedures. We now provided the R-scripts to run the whole analysis.

and that the R-squared of the best model seems to be really low (<3%), which question the robustness of the results (at least for the shearwater data, and I would urge The authors to present R squared for the others species as well).

**The authors:** This was a misunderstanding. In general, egg volume variability in birds depends on genetic effects (female size; >80%), approximately a 9% depends on food intake and around 11% depends on other factors (see Christians 2002 cited in the text). What we assessed is how the mean egg volume changes over the years and how much of this variation is due to environmental factors (large-scale vs local). For example, if the total volume of an egg is 75 cm^3^, approximately 60 Cm^3^ depends on the size of the female, the remaining 15cm^3^ depends on environmental conditions (e.g. food intake). We aimed to explain the inter-annual variations of those 15cm^3^ considering environmental conditions measured at different spatio-temporal scales. Results show that there is a strong correlation between the egg volume observed and the egg volume predicted by the models (see e.g. Figures 3, S15, S16 and S17). R-squared are all above the 0.59.

Moreover, The authors did not present the fit of the models (relationships between egg volume and covariates for the best models)

**The authors:** We are not sure to have understood this comment. We assessed the fit between the mean egg volume predicted and the mean egg volume observed, which is 59-79% depending on the species (see Results section and S15, S16 and S17).

and did not say whether the basic assumptions were verified (linearity, normality, homogeneity independence), which also makes it difficult to judge the quality of the fit of their results.

**The authors:** We agree. We have now added more information on model performances in the manuscript. The tests used to check these assumptions can be also found in the R-scripts.

Instead The authors seem to have used an inappropriate formula, originally adapted to survival models made for capture history data (L190-191). This type of data does not seem to be part of the study design. In case I am wrong, this would mean the entire Methods section should be completely rewritten to better explained how modeling where conduced. I suggested R packages and alternatives approaches to analyze their data, to assess goodness of fit and the relative importance of each predictor (R code attached and details comments below). I also questioned whether mixed effects models could/should be used to control for potential inter-annual variability.

**The authors:** We have used these formulas to assess the proportion of the temporal variance explained by the best models. On the other hand, the correlations shown in figures S15, S16 and S17 also support these results.

In conclusion, although I must say that the conclusion brought by The authors, of the importance of availability and accessibility of resources is very interesting to me, I was unfortunately not convinced by the analyses, and I hope the R code that I shared could be helpful to improve the manuscript. Below I provide detailed comments that I hope will be helpful:

Title
L1: “accessibility of” instead of “to” I think

**The authors:** We have changed that.

L3: Given the introduction and especially the discussion very focused on seabirds I would change “animals” to “seabirds” instead, or at least to “marine animals” or “marine predators”

**The authors:** Thank you, we agree and we have changed it accordingly.

Abstract
L20: “long-term” please specify how many years (at least in average) directly in the abstract, please also specify studied regions, species names and sample size.

**The authors:** We agree. However the Abstract already has 225 words and we would like to stay well within the limits fixed by the journal. These details can be found in the Methods section.

L24: “competition” please specify inter- and or intra-specific competition?

**The authors:** This information is also detailed in the Methods section. We believe that including this information in the Abstract would not improve it.

L26: “predictive power” estimated with cross-validation was not preformed in this study, perhaps “goodness of fit” instead?

**The authors:** We assume the reviewer refers to the use of cross-validation to predict the error in Generalized Linear Models. Here we refer to the predictive power of the models.

L30: please specify if “detectability” corresponds to “foraging conditions” and “accessibility” to “competition” (by splitting the bracket). If it is not the case, please consider to add in bracket that both are influenced by same processes (foraging conditions + competition)

**The authors:** In this sentence we considered that foraging conditions may influence both, detectability and accessibility of food. However, we agree that competition has more to do with food accessibility so we have changed this sentence.

Introduction
L54: “marine” top predators would be better I think

**The authors:** We agree and we have added “*marine*” to the sentence. However, in this study, we use large-scale climatic indices and long-term data on seabirds' egg volume. For this reason, it is also very important to keep in mind that these seabirds are “*long-lived and long-ranged marine top predators*”.

L67: more details of different “life histories” traits would required in my opinion
**The authors:** In this study, we do not use data on ecological parameters at the individual level, because they were not fully available. However, some species-specific life-history traits that are important for the study are provided through the manuscript and the supporting information (see e.g. Table S4).

L80-83: “we assess the influence of i) [...] ii) [...] as predictors of...” please reformulate, this sentence is not correct

**The authors:** We agree and we have modified the sentence accordingly.

L83-84: please provide here a clear definitions of detectability and accessibility directly in the introduction, as they are key concepts of the study.

**The authors:** Detectability and accessibility are indeed key concepts. We described these terms in the Methods section when we refer them to the covariates considered.

In my opinion, they are not interchangeable, and therefore should also be considered separately in the text, whereas they are always used a combination (e.g. L136, L211, L268, L272, L274, L281).

We agree. We used these terms together throughout the text because some of the covariates used in the models influence both, detectability and accessibility of food. We do not mean that they are interchangeable. Simply they should be ‘considered’ together.

I invite The authors to cite Matthiopoulos 2003, a seminal article on accessibility in ecology.

**The authors:** Thank you for the reference. We have cited this article in the manuscript and added it to the list.

L85-87: There is a clear lack of predictions/expectations of how effects on egg volume should change differently depending on species foraging strategies, life histories, and population densities. One idea would be to summarize these in a summary table...

**The authors:** We agree. These issues have been discussed in de Discussion section. See also Tables S4 and S5.

Methods
L98: “temporal variance” it is not clear to me how variance will be then used in the analyses. From the data and results, only the average egg volume seems to have been analyzed, but not the change in variance over time?

**The authors:** The change in egg volume in relation to an average value has a clear temporal components. Here by temporal variance we refer to the changes of the mean egg volume over the years. In general, less than 9% of the egg volume in birds can be explained by food (See e.g. Christians 2002) as most of the total egg volume (around 80%) depends on genetic-related factors (e.g. female size) which here we were not be able to account for. Our aim is not to assess the influence that covariates have on the total egg volume. We use models to assess how the proportion of the egg volume that depends on environmental factors (the 9% mentioned above) changes over the years.

L104: “eggs volume were measured” this was done before calculating “temporal variance” I guess (cf. Precedent comment)? If yes, please consider re-ordering

**The authors:** See comments above.

L109: I would have deleted it if it wasn't “necessary”, plus the paragraph on ethics right after is already long enough.

L112-119: This ethic paragraph could shorten, I think.

**The authors:** We agree. This is because of the many government institutions that have to be mentioned.

L120: “Predictors” the term “covariates” is also used later, it would be better for the reader to choose a term and remain consistent in the manuscript.

**The authors:** We partially agree. The term covariates refers to the set of values of one or more (co)variables that are used as predictors (idependent variables) of the egg volume. In this case “predictors” can refer to a set of covariates and it is used to inform the reader that we used a combination of several covariates. Technically speaking a predictor can be an interaction effect, while a covariates are only a single variable (rather than a combination of two or more covariates). This difference depends of course on the sentence. For example the term predictors in the title “Predictors of the egg volume” is more informative than “Covariates of the egg volume”.

L130: Paragraph title could be renamed as “Accessibility vs. Detectability” for clarity (with a subtitle perhaps)

**The authors:** This would not be fully correct because accessibility also involves competition, which is described in another section.

L131: I don't quite see what these "physical barriers" could be that could for example prevent access but not detection?

**The authors:** There are many examples. The wave height for example can reduce the number of successful attempts in Sandwich terns (see also references in the text).

It can also be argued that birds must first access foraging areas based on, for example, winds, distances to colonies, and forager densities, and then, on a finer scale, engage in a area restricted search (ARS), where detectability plays a more important role, based on, for example, water turbidity, wave height etc (see Weimerskirch 2007).

**The authors:** When we use the term "accessibility", we are not only referring to the foraging areas but also to the food itself. According to our approach, seabirds must first detect food (prey) and then try to access it.

L136: “detectability and accessibility” it seems to be considered interchangeable here, whereas they are two distinct processes with different scales and potentially influenced by different factors. More general and theoretical references could be cited here.

**The authors:** See the answer to the similar comment above. These two terms are different of course, but sometimes a given covariate can influence both.

L139: It is very nice to investigate cumulative effects! We can also look at the variance of the covariates instead of the mean values (cf. Stenseth et al 2002)

**The authors:** We agree and it is an interesting comment. We would like to investigate the variance as well as the predicted trajectories of the covariates provided by current scenarios of future oceanographic changes. However, we will leave this interesting aspect for a future work.

L146: Why “early” is not clear to me, this should be defined in the ‘Field data’ paragraph

**The authors:** We said ‘early’ because this is the period when seabirds mobilize reserves used in the formation of eggs but we ignore the exact timing for each species or mechanisms involved. We used a general term to mean ‘before’.

L148: “breeding pairs numbers” could be a poor indicators of inter- and intra-specific competition, as densities at sea can vary according to many factors (social behavior, environmental productivity, variability and predictability, etc) it might be better idea to use distance to the colony as a proxy of competition (cf. Wakefield et al. 2011,

**The authors:** We agree. Competition and behaviourl aspects of foraging at sea are interesting issues to explore. Unfortunately we do not have enough telemetry data for each species to investigate this point.

L159: Does “Per capita” refer to per individual, per species, per fisheries boat? Please reformulate

**The authors:** The Latin meaning of the term “*per capita*” is “per head”. Therefore, it refers to individuals.

L163: The "CPUE" is actually a proxy of prey available to seabirds, but this would need better justification (i.e., reference materials) because it seems to me that what is caught by fisheries does not necessarily reflect what is available to seabirds, unless one assumes that catches are proportional to fish stocks

**The authors:** Catch per unit of effort (CPUE) is an indirect measure of the abundance of a target species widely used in fishery stock assessment. Using a proxy like this is the best option in studies that consider large temporal and spatial scales.

and that fisheries and seabirds forage in the same areas (in which case the assumptions would need to be clearly stated)

**The authors:** Datasets used to estimate CPUE correspond to the same areas were seabirds forage during the breeding season when foraging areas are more restricted (see Methods section).

L170: Why “horsepower” could be considered as a good proxy for fisheries discarded should be clearly explained (readers should understand without looking a the reference is not enough).

**The authors:** We agree. Now we have added more details in this regard.

L170: “Egg volume” and not its “temporal variance” is analyzed instead of what it is said L98.

**The authors:** We agree. Thank you for pointing this out. There is a mistake in L98. We have now changed the sentence “*we recorded the temporal variance of the annual mean volume of the modal clutch*” by “*we recorded the annual mean volume of the modal clutch*”

L178: “generalized linear models” the family used to model egg volume is not given. I am assuming that the gaussian family with an identity link function. In that case the authors in fact used a simple (multiple) linear regression and not a GLM (or maybe a GLS if they used more complex variance structure cf. Zuur et al. 2009).

**The authors:** Correct. We used Gaussian family and identity link. The multiple linear regressions (MLR) are indeed a particular case of GLM. However in MLR parameters are least-squares estimates. See the answer to a similar comment above. In this case the two terms are correct, but GLM also inform on how parameters are estimated.

L179: “modal clutch” please define what “modal” means

**The authors:** We agree and we have now improved this sentence accordingly.

L179-181: This sentence is completely unclear to me. Did you standardized all variables using center=T and scale=T with the scale function in R (so that means=0 and standard deviation=1)?

**The authors:** No, we first scaled covariates (only those needing to be scaled) by dividing by 1000. Then we centered these variables by substracting the mean to each value which is equivalent to the using “center=T” in R. Now we have modified this sentence for better understanding.

Dividing by 1000 is not necessary in my opinion.

**The authors:** Using the function “scale=T” is only another way to scale data.There are many ways to scale data. Dividing by 1000 is simply another way to do it.

Also, I tried to fit the best model for the shearwater using the data available online, and to be able to retrieve the same estimates presented in supplementary (Table S12), I had to use the unscaled data (the raw data, please see attached R code). Could the authors clarified this point?

**The authors:** We think the reason you have to downscale the data to get the same results is that the data available online has already been scaled. Now the original scripts are also available for review. Observe that when the name of our covariates start with the term "ScalStd..." like e.g. "ScalStdTrawlHp" or "ScalStdCPUE_Sardine", this means that these covariates have already been scaled and standardized.

L183: “collinearity” Variance Inflation Factors should also be investigated (cf. Zuur et al. 2009)

**The authors:** We agree. We used cross correlations to assess for collinearity and we have not considered together correlated covariates in the same model. Now we have also performed a VIF analysis for best models as suggested. Considering a threshold value of 3 as in Zuur et al. 2010, and there is no evidence of collinearity as in the cross-correlation methods. VIF analysis are also included in the scripts. In models with interactions, interaction terms were also centered (using center=T) as suggested by Robinson and Schumacker 2009. We have also added an explanation of this procedures in the manuscript as follows:

“*For the best models, we also run tests to check the variance inflation factors (VIFs). Only models were all VIF values were <3 were considered [48]. To check VIFs in models with interaction terms we first centered these covariates as suggested by [49].”*

We have also added these two references:

48. Zuur, A. Leno, E.N. and Elphick, C.S. A protocol for data exploration to avoid common statistical problems. Methods Ecol. Evol. 2010; 1: 3-14. <http://www.respond2articles.com/MEE/>
49. Robinson, Cecil & Schumacker, Randall. (2009). Interaction Effects: Centering, Variance Inflation Factor, and Interpretation Issues. Multiple Linear Regression Viewpoints. 35.

L185: The authors should explained more (by making clear predictions in the introduction) what make “ecological sense” as models tables as they stands look the results of arbitrary choices.

**The authors:** We disagree. Models have been selected using realistic hypotheses on what may influence ecological process or species-specific characteristics. We wrote “…*ecological sense according to species-specific diet and foraging strategies*”.

L189: “The proportion of total annual variance” is unclear to me, the formula would required a reference from a statistical paper or book.

**The authors:** Please, see comments above and the last paragraph of the Methods section.

L190-191: The terms "constant model" and "time-dependent model" make no sense to me here, it seems that the authors are trying to use a survival model with a notation made for capture history data. If I have misunderstood, much more detail should be provided on the design of the study (are the marked and known birds or nests monitored each year? see how the data are presented, for example, in Harris et al. 2005).

**The authors:** We partially agree. Model notation (and model description) is in many cases subjective. The important point is that notation is explained for the readers to understand the models. We did it here and we used a notation similar to the GLM syntax for those reader familiar with it. Also note that in the suggested reference by Harris et al. 2005 models are described as “..constant and fully time-dependent models” (pag 291). This description refers indeed to survival models which are logistic regressions (another particular case of GLM).

L191-192: Since I assume the authors used a GLM with a Gaussian distribution, this is equivalent to a linear model for me, so this formula doesn't make sense to me here. And the R-squared should be provided. The relative proportion of variance explained by each predictor for the best model could then be given, in order to assess their relative importance (cf. rwa or relaimpo R packages, see Grömping 2006).

**The authors:** See the answer to a similar comment above about GLM vs linear regression. We assessed the fit using predicted vs observed values. Equivalences of R-squares are given in the text.

L192: The classical diagnostic plots to check for models assumptions should be provided in supplementary material and or at least to be said to have been checked. Here the list of assumptions (also worked for GLMs):
1.Linearity of the data
2.Normality of residuals
3.Homogeneity of residuals variance
4.Independence of residuals error terms

**The authors:** We agree. We have now modified the text. The functions used for model diagnostics are also found in the scripts.

L193: It is particularly important to test the linearity of the relationships, because nonlinear relationships are widely reported in ecology (such as the bell shape) and easily verified (by the use of polynomials or generalized additive models).

**The authors:** We agree. Idem to previous response.

L193: As I understand it, bird identity is not available for the study, which would explain why mixed-effects models cannot be used. However, given that several data are nested by year, I wonder if at least the year should not be used as a random structure (it seems that several studies on egg size change have done this, e.g. Kvalnes et al. 2013, Verhoeven et al. 2019).

**The Authors:** We agree in theory, however “year” as a fixed factor was significant and this was the variability we wanted to explain using the covariates. Having both types of variables (year and covaraites) into the model was redundant.

Results:
L198: Figure 3 presents the time series of egg volume whereas the we are expecting to see how the drivers are correlated to egg volume. Outputs of best models should thus be given (I would encourage the authors to look at the R car package and the avPlots function, and the ggeffects R package with ggpredict function, please see R code and references attached).

**The Authors:** Figure 3 shows the observed values of egg-volume over time and those predicted by the best model. In this sense it can be seen as a graphical illustration of the fit of the model. This is explained in the figure text.

L199: “decreased the egg volume” this is a too strong assertion since this is only based on correlation,

**The Authors:** The model constrains the expected value to a linear trend. Whether this result an increasing or decreasing trend depends on the sign of the linear predictor. In this case it was predicted a decreasing trend based on statistical inference.

it is very likely that these are the result of indirect effects.

**The Authors:** We disagree. Several studies have shown that wave height has a direct and statistically significant effect on seabirds foraging success (see reference list).

L202: “separately” this corresponds to which model ? (model 4 for wNOA I guess, but I cannot find local condition only models?)

**The Authors:** We agree. Now we have included these three models (one per species) with local conditions only (see Model 3 in Tables 1 and S1; Model 10 in Tables 2 and S2 and Model 4 in Tables 3 and S3).

I would rather use the best model and to partition the variance per predictor.

**The Authors:** We understand. It is a slightly different way to show the results. We did specify the variance explained by the different set of predictos (local vs large-scale). We think it is a more direct way to inform readers, considering the starting hypotheses.

L202: “total annual variance” I did not understood how authors managed to calculate this, in case of GLM the proportion of explained variance should be 1-deviance/null deviance. But here as gaussian regressions have been used, the R-squared should be reported. I did fit the best shearwater model with the data available and the R-squared is rather low with only 3% (see code attached), I also tried to plot the regression lines, and it appears that slopes of the relationships looks very weak. I am thus wondering if the effect found by the authors is rather anecdotal, and I would encourage to explore new covariates.

**The Authors:** See answer to a similar comment above. We had explained the rational and each step of the analysis in the manuscript and in the supplementary information for a better understanding.

Table 1: I would be more interesting to me to present effect sizes of the models (estimates e.g. Table S12) rather than (incomplete) model selection of Tables 1, 2, and 3. I think providing complete model selection tables in supplementary is already enough. Pvalues or at least 95% Confidence Intervals should be provided however for the estimates tables, with R-squared (e.g. Table S12).

**The Authors:** We disagree and we consider the results have been presented following the objectives of the study.

Table S2: few models were below dAIC<2 and thus model averaging should be conduced I think for these Sandwich tern models.

**The Authors:** We agree and we mentioned that some models had similar AIC values although they include extra variables. However in comparing nested models these variables act as a “pretending varaibles” (see Anderson, D.R. 2008. Model Based Inference in the Life Science: a primer on evidence, ed Springer pg 65). They do not add any explanatory power (a small drop in deviance), but, because nested, they necessarily result in a similar AIC value. We wrote that:

“*Models 2 to 5 including the additive effect of the interspecific competition exerted by Yellow-legged gulls, seawater turbidity, or the statistical interaction with the 3^rd^ quartile winds, had similar explanatory power to Model 1 (i.e. ∆AIC values < 2; Table 2 and S2 Table). However, both the interaction and the additive terms in models 2 and 5 act as pretending variables. Pretending variables occur when after adding a new variable in a model, a ΔAIC~2 is obtained but, the deviance does not decrease. [51] and should not be considered further (Appendix B in [52]).”*

Discussion

In my opinion the Discussion is too focused on the results and studied species, and therefore failed to generalize over a broader range of species, systems and life histories. There is also a lack of comparisons with studies that also investigated changed in egg volume such as Barrett et al. 2012, Bennett et al. 2017, Tomita et al. 2009, Verhoeven et al. 2019.

**The Authors:** We agree. Our first goal was to investigate the role of large-scale and local variables as linear predictors of interannual variations in egg volume. We are aware that the literature on egg size is vaste but we wanted to concentrate on these predictors.

These results are only correlative, could the authors think and suggest ways / experiments to answer this drawback?

**The Authors:** This is a nice and important question. Indeed our study is correlative and it is not possible to provide long-term experimental data in these species. In same cases, for example, the colony was made by thousands of breeding pairs. Indeed, most studies assessing the effect of large-scale climatic indices on ecological parameters are based on correlations as the mechanisms behind these processes are often too complex to be assessed empirically. In our study, we first considered empirical studies explaining the most elemental factors influencing these mechanisms (influence of food intake on egg volume, the influence of waves and visibility on foraging success etc.) and then, we assumed that this could be transferred to a broader spatial scale. For example, it is known that the egg volume partially depends on food intake. On the other hand, it is quite clear that food intake must strongly depend on two key factors: food abundance and foraging conditions. So, it makes sense to think that foraging conditions influence food availability to seabirds by modulating its detectability and accessibility. Several studies have measured empirically the influence of foraging conditions (wave height and/or visibility) on seabirds foraging success. Large-scale experiments at population level are opportunistic (e.g. a fishing moratory or the closure of a local open air landfill) and cannot be conducted on a long-term. It would certainly be interesting to have more experimental data.

Data
No readme.txt, and no codes, which make the analyses hardly reproducible. For example are the variables already scaled? What are the units of each variable? What family and link function were used for the GLMs? How models within models selection table were chosen?

**The Authors:** We agree. Now the original R-scripts with all the analyses are fully available.

Thank you on behalf of the authors for helping to improve this study.

References mentioned in this review

Barrett, R., Nilsen, E. & Anker-Nilssen, T. (2012). Long-term decline in egg size of Atlantic puffins Fratercula arctica is related to changes in forage fish stocks and climate conditions. Mar. Ecol. Prog. Ser., 457, 1–10.

Bennett, J.L., Jamieson, E.G., Ronconi, R.A. & Wong, S.N.P. (2017). Variability in egg size and population declines of Herring Gulls in relation to fisheries and climate conditions. ACE, 12, art16.

Catry, P., Lemos, R.T., Brickle, P., Phillips, R.A., Matias, R. & Granadeiro, J.P. (2013). Predicting the distribution of a threatened albatross: The importance of competition, fisheries and annual variability. Progress in Oceanography, 110, 1–10.

Grömping, U. (2006). Relative Importance for Linear Regression in R : The Package relaimpo. J. Stat. Soft., 17.
Harris, M., Anker-Nilssen, T., McCleery, R., Erikstad, K., Shaw, D. & Grosbois, V. (2005). Effect of wintering area and climate on the survival of adult Atlantic puffins Fratercula arctica in the eastern Atlantic. Mar. Ecol. Prog. Ser., 297, 283–296.

Kvalnes, T., Ringsby, T.H., Jensen, H. & Sæther, B.-E. (2013). Correlates of egg size variation in a population of house sparrow Passer domesticus. Oecologia, 171, 391–402.

Matthiopoulos, J. (2003). The use of space by animals as a function of accessibility and preference. Ecological Modelling, 159, 239–268.

Tomita, N., Niizuma, Y., Takagi, M., Ito, M. & Watanuki, Y. (2009). Effect of interannual variations in sea-surface temperature on egg-laying parameters of black-tailed gulls (Larus crassirostris) at Teuri Island, Japan. Ecol Res, 24, 157–162.

Verhoeven, M.A., Loonstra, A.H.J., McBride, A.D., Tinbergen, J.M., Kentie, R., Hooijmeijer, J.C.E.W., et al. (2020). Variation in Egg Size of Black-Tailed Godwits. Ardea, 107, 291.

Wakefield, E.D., Phillips, R.A., Trathan, P.N., Arata, J., Gales, R., Huin, N., et al. (2011). Habitat preference, accessibility, and competition limit the global distribution of breeding Black-browed Albatrosses. Ecological Monographs, 81, 141–167.

Weimerskirch, H. (2007). Are seabirds foraging for unpredictable resources? Deep Sea Research Part II: Topical Studies in Oceanography, 54, 211–223.

7. PLOS authors have the option to publish the peer review history of their article ([what does this mean?](https://journals.plos.org/plosone/s/editorial-and-peer-review-process#loc-peer-review-history)). If published, this will include your full peer review and any attached files.


If you choose “no”, your identity will remain anonymous but your review may still be made public.


**Do you want your identity to be public for this peer review?** For information about this choice, including consent withdrawal, please see our [Privacy Policy](https://www.plos.org/privacy-policy).

Reviewer #3: No

Reviewer #4: No

[NOTE: If reviewer comments were submitted as an attachment file, they will be attached to this email and accessible via the submission site. Please log into your account, locate the manuscript record, and check for the action link "View Attachments". If this link does not appear, there are no attachment files.]

While revising your submission, please upload your figure files to the Preflight Analysis and Conversion Engine (PACE) digital diagnostic tool, <https://pacev2.apexcovantage.com/>. PACE helps ensure that figures meet PLOS requirements. To use PACE, you must first register as a user. Registration is free. Then, login and navigate to the UPLOAD tab, where you will find detailed instructions on how to use the tool. If you encounter any issues or have any questions when using PACE, please email PLOS at [figures@plos.org](javascript:void(window.open('/imp/dynamic.php?page=compose&to=figures%40plos.org&popup=1%27,%27%27,%27width=820,height=610,status=1,scrollbars=yes,resizable=yes%27))). Please note that Supporting Information files do not need this step.

*In compliance with data protection regulations, you may request that we remove your personal registration details at any time.* [*(Remove my information/details)*](https://www.editorialmanager.com/pone/login.asp?a=r)*. Please contact the publication office if you have any questions.*
